# Compound genetic etiology in a patient with a syndrome including diabetes, intellectual deficiency and distichiasis

**DOI:** 10.1186/s13023-022-02248-2

**Published:** 2022-02-28

**Authors:** Lauriane Le Collen, Brigitte Delemer, Marta Spodenkiewicz, Pascale Cornillet Lefebvre, Emmanuelle Durand, Emmanuel Vaillant, Alaa Badreddine, Mehdi Derhourhi, Tarik Ait Mouhoub, Guillaume Jouret, Pauline Juttet, Pierre François Souchon, Martine Vaxillaire, Philippe Froguel, Amélie Bonnefond, Martine Doco Fenzy

**Affiliations:** 1 Department of Endocrinology Diabetology, University Hospital Center of Reims, Reims, France; 2 Inserm/CNRS UMR 1283/8199, Pasteur Institute of Lille, EGID, Lille, France; 3 University of Lille, Lille, France; 4 Department of Genetic, University Hospital Center of Reims, Reims, France; 5 Faculty of Medicine of Reims, CRESTIC EA 3804, University of Reims Champagne Ardenne, Moulin de La Housse, BP 1039, 51687 Reims Cedex 2, France; 6 Laboratory of Hematology, University Hospital Center of Reims, Reims, France; 7 Departement of Genetic, 1 rue Louis Rech Dudelange, 3555 Luxembourg, Luxembourg; 8 Diabetology, Medipole Lyon-Villeurbanne, Lyon, France; 9 Department of Pediatric Diabetology, University Hospital Center of Reims, Reims, France; 10 Faculty of Medicine of Reims, EA 3801, URCA, Reims, France

**Keywords:** Childhood onset diabetes, Genotype–phenotype relations, Genetic disorders, Genetic analysis, Intellectual disability, Wolfram syndrome

## Abstract

**Background:**

We studied a young woman with atypical diabetes associated with mild intellectual disability, lymphedema distichiasis syndrome (LDS) and polymalformative syndrome including distichiasis. We used different genetic tools to identify causative pathogenic mutations and/or copy number variations.

**Results:**

Although proband’s, diabetes mellitus occurred during childhood, type 1 diabetes was unlikely due to the absence of detectable autoimmunity. DNA microarray analysis first identified a de novo, heterozygous deletion at the chr16q24.2 locus. Previously, thirty-three pathogenic or likely pathogenic deletions encompassing this locus have been reported in patients presenting with intellectual deficiency, obesity and/or lymphedema but not with diabetes. Of note, the deletion encompassed two topological association domains, whose one included *FOXC2* that is known to be linked with LDS. Via whole-exome sequencing, we found a heterozygous, likely pathogenic variant in *WFS1* (encoding wolframin endoplasmic reticulum [ER] transmembrane glycoprotein) which was inherited from her father who also had diabetes. *WFS1* is known to be involved in monogenic diabetes. We also found a likely pathogenic variant in *USP9X* (encoding ubiquitin specific peptidase 9 X-linked) that is involved in X-linked intellectual disability, which was inherited from her mother who had dyscalculia and dyspraxia.

**Conclusions:**

Our comprehensive genetic analysis suggested that the peculiar phenotypes of our patient were possibly due to the combination of multiple genetic causes including chr16q24.2 deletion, and two likely pathogenic variants in *WFS1* and *USP9X*.

**Supplementary Information:**

The online version contains supplementary material available at 10.1186/s13023-022-02248-2.

## Background

Type 2 diabetes is a non-autoimmune, multifactorial metabolic disorder characterized by chronic hyperglycemia resulting from impaired insulin secretion and altered action of insulin [1]. Type 2 diabetes depends on various environmental components while having a high heritability ranging from 40 to 70% [2]. Apart from common type 2 diabetes that affects most cases with non-autoimmune diabetes, there are atypical, monogenic forms of diabetes which are usually rare and severe, with an early onset. Pathogenic mutations in more than 40 genes have now been described in patients with monogenic diabetes. They cause various conditions including neonatal diabetes, maturity-onset diabetes of the young (MODY), and diabetes-associated syndromes (e.g. Wolfram syndrome due to variants in *WFS1*, Wolcott–Rallison syndrome) [3]. In Wolfram syndrome (OMIM #222300), juvenile-onset- diabetes mellitus can be isolated or associated with more complex phenotypes including optic atrophy, diabetes insipidus, and deafness. Several genes linked with monogenic diabetes are actionable (such as *KCNJ11 *[5],* ABCC8* [5], * GCK* [5], * HNF1A *[5], * HNF1B* [5, 6] or *HNF4A* [5]), implying a substantial change in care for the carriers of a pathogenic variant (such as specific therapeutic management and monitoring) [4, 5]. Therefore, the genetic diagnosis of patients with a suspicion of monogenic diabetes has become crucial.

Here, we performed karyotyping, DNA microarray, and whole-exome sequencing in order to detect point mutations and/or copy number variations (CNVs) putatively causing a complex syndrome (including an atypical diabetes and polymalformative syndrome) in a young woman.

## Results

### Phenome of the case report

A 17-year-old girl was referred after a pediatric follow-up for a non-autoimmune diabetes and a mild intellectual disability. She was from a non-consanguineous family and had two healthy siblings.

On the maternal side, her mother is an only child, and presented with mild dyscalculia and dyspraxia. None other members of this part of the family had an intellectual disability.

On the paternal side, her father has had type 2 diabetes since age 50. There was no history of diabetes in any of the three patient’s uncles. Her paternal grandmother presented with a late isolated cataract (from age ~ 80).

She was born at 41 weeks gestation after an uneventful pregnancy. Her birth weight was 3.620 kg (67th percentile), her size was 51 cm (67th percentile), and had a normal head circumference (85th percentile). Diabetes was diagnosed at age 7 while the young girl suffered from sudden polydipsia, polyuria, and unexplained weight loss (− 1.50 kg). At hospital admission, venous glycaemia level was 5.34 g/L, with 2 + in urinary ketone testing, glycated hemoglobin A1c (HbA1c) was very high (12.8%; Normal range: < 6.5%) and C-peptide was low (0.27 ng/mL; Normal range: 1.2–4.5 ng/mL). The venous pH, blood sodium and potassium levels were normal, with alkaline reserve at 26 mmol/L. Neither islet autoantibodies, nor anti-insulin antibodies were present. Anti-GAD, anti-IA2, anti-ZNT8 antibodies were not initially analyzed. Insulin therapy was initiated and the patient was regularly followed up. HbA1c had remained between 7.5% and 8.5% overtime without episodes of ketosis or severe hypoglycemia. At the last clinical investigation at age 24, no chronic complications of diabetes were reported. Anti-GAD, anti-ZnT8 and anti-IA2 antibodies were still negative. Pancreas imaging did not show any pancreatic atrophy.

**Fig. 1:**
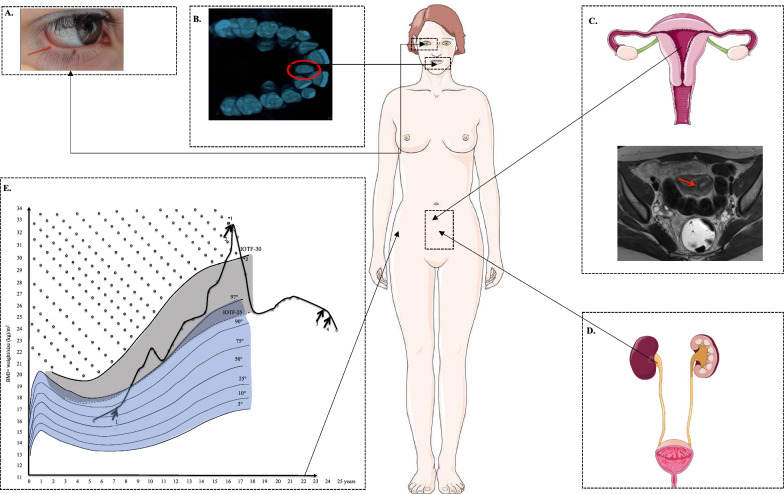
Clinical features of the patient—Polymalformative syndrome including distichiasis (red arrow in **a**), palatine tooth (circled in red in **b**), uterine septum (red arrow in **c**), vesicoureteral reflux (**d**) and overweight (**e**). In part E we reported the increase in insulin requirement during childhood (arrow #1: 1.1 ui/kg) followed by the decrease in insulin requirement after the addition of metformin (arrow #2: 0.7 UI/kg). We show the addition effects of DPP4 inhibitors (arrow #3), and GLP1-RA (arrow #4). The dark area shows the values corresponding to overweight, while the area with points shows the values corresponding to obesity

We observed a severe gain of weight at age 8, concomitant to insulin treatment, reaching the 97th percentile of body mass index (BMI) (Fig. 1). Weight gain persisted throughout puberty. No endocrine causes of obesity were found, but low limbs swelling were regularly reported by the patient suggesting lymphedema. At age 16, the patients suddenly needed more insulin (1 UI/kg)*.* Furthermore, metformin (1 g per day) was added to insulin injections (Fig. 1). This additional treatment led to an initial and persistent weight loss and to a 40% reduction in insulin doses. Then, we have tested the action of the incretin pathway agonists on diabetes control and insulin requirement. First, a dipeptidyl peptidase 4 (DPP4) inhibitor was added to her insulin injections, and we observed a decrease in HbA1c after 3 months (8.9 to 7.9%) and 1 kg weight loss (**Fig. ****1**)*.* Then, we have switched from prandial insulin to glucagon-like peptide-1 receptor agonist (GLP1-RA) (Liraglutide 1.8 mg per day) associated with basal insulin only*.* The patient has further lost 3.6 kg (Fig. 1).

In addition, after ruling out deafness, we assessed psychomotor retardation of the patient during her childhood. She used sentences at age 6. At age 8, the intelligence quotient (IQ) test showed a mild intellectual disability (WISC III test: total IQ 68). Moreover, at age 5, the patient was diagnosed with a bilateral distichiasis in the lower and superior eyelids, complicated with keratitis, which required surgery followed by laser and recurrent epilation. She also had dental malposition and palatine tooth requiring a surgery during her childhood. At age 8, she had diurnal enuresis. Urodynamic examinations showed that the patient also had vesicoureteral reflux with retro-urethral meatal stenosis and overactive bladder. Kidney ultrasound showed renal calyceal, hypotonia and duplex kidney. Despite anticholinergic drugs, the injection of botulinum toxin was required to manage bladder instability. After puberty, at age 13, the patient suffered from painful dysmenorrhea. Pelvic ultrasound and magnetic resonance imaging showed that the patient presented with uterine septum, which required surgery*.*

### Genetic investigations

**Fig. 2:**
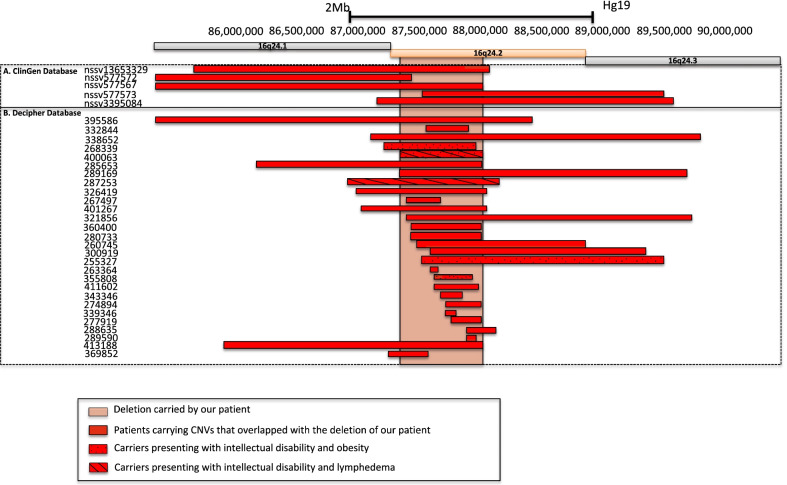
Pathogenic or likely pathogenic deletions encompassing chr16q24.2, which were found in 33 patients from Decipher and ClinGen databases

**Fig. 3:**
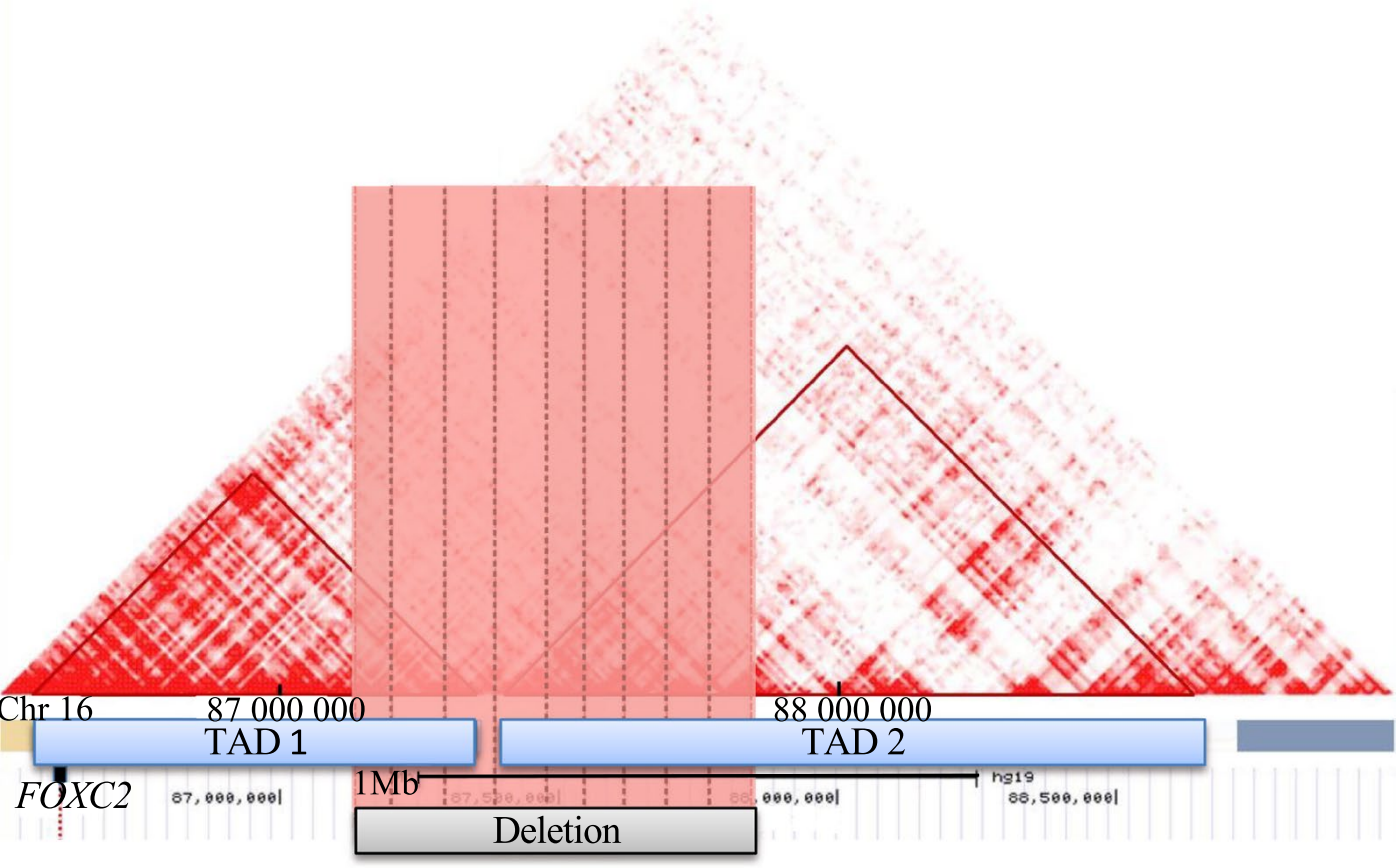
Identification of two TADs (from fetal brain) encompassing the deleted region at the 16q24.2 locus. Deletion found in the patient is represented by a grey rectangle. TAD 1 and TAD 2 are represented by triangles. The deleted region, containing part of the TADs, is highlighted in red

The karyotype was normal. Through array comparative genomic hybridization (CGH) performed in the trio (proband *versus* both parents), we found a de novo heterozygous deletion of 693 kb (chr16q24.2; 16: 87,152,792–87,845,741 [hg19]) in the proband. The deleted region includes six protein-coding genes (Table 1)*.* This result was confirmed by real-time PCR. According to the Human Gene Mutation Database (HGMD), none of these genes were consistently found to be linked with monogenic forms of diabetes, obesity, kidney disorders, intellectual deficiency, lymphedema and/or distichiasis. However, other deletions encompassing chr16q24.2 have already been reported in 33 patients according to ClinGen and Decipher databases (Fig. 2 and Table 2)*.* Among them, 25 patients presented with intellectual disability (Fig. 2 and Table 2). It is noteworthy that four patients shared intellectual disability, obesity or lymphedema as observed in our patient (Fig. 2 and Table 2). Furthermore, we observed that *FOXC2* which is linked with lymphedema distichiasis syndrome (LDS) (Additional file 1: Table S1) was located 550 kb downstream to the chr16q24.2 deletion. This gene encodes a transcription factor belonging to the forkhead family. Using HiC data obtained from human fetal brain, we found that the chr16q24.2 deletion encompassed two subsequent topologically association domains (TADs) (Fig. 3). *FOXC2* was located in the first TAD (Fig. 3). Therefore, we have hypothesized that the deletion might disrupt the transcriptional network of *FOXC2*, possibly leading to lymphedema in the carriers of chr16q24.2 deletion [7].

**Table 1:** List of genes and their transcripts included in the chr16q24 deletion carried by the proband (UCSC Genomic Institute. UCSC Genomic Institute (University of California Santa Cruz)

Gene	Transcript	Chr	Tx-Start [hg19]	Tx-End [hg19]	Exon Count	Strand	Function	Disease
*C16orf95*	NM_001195124	16	87,336,420	87,350,998	7	−	Unknown	Unknown
*FBXO31*	NM_024735	16	87,360,592	87,417,382	9	−	Degradation via Skp1-Cul1- Fbox protein complex; cell cycle regulator [33]	Intellectual disability autosomal recessive
*MAP1LC3B*	NM_022818	16	87,425,941	87,438,380	4	+	Autophagy pathway [33]	Unknown
*ZCCHC14*	NM_015144	16	87,439,853	87,526,630	13	−	Regulates tumor progression [34]	Unknown
*JPH3*	NM_001271604	16	87,636,113	87,638,254	2	+	cross talk between cell surface and intracellular ion channels by junctional complexes (OMIM* 60,526)	Huntington’s Disease Like 2
*KLHDC4*	NM_017566	16	87,741,417	87,799,592	12	−	Unknown	Unknown

*Chr* Chromosome, *Tx* transcription

**Table 2:** Pathogenic or likely pathogenic deletions encompassing chr16q24.2, which were found in 33 patients from Decipher and ClinGen databases

Sample's ID	Position (Hg19)	Size (Mb)	Pathogenicity	Phenotypes
nssv577572	chr16: 83,912,597–87,257,444	3.3	Pathogenic	Developmental delay and/or other significant developmental or morphological phenotypes
nssv13653329	chr16: 85,491,404–87,883,528	2.4	Pathogenic	Premature birth, respiratory distress, obsolete malformation of the heart and great vessels
nssv577567	chr16: 78,738,172–87,852,948	9.1	Pathogenic	Low-set ears
nssv577573	chr16: 87,340,135–89,335,487	2	Pathogenic	Developmental delay and/or other significant developmental or morphological phenotypes
nssv3395084	chr16: 86,983,712–89,402,222	2.4	Pathogenic	Autistic behavior, cleft palate, delayed fine motor development, delayed gross motor development, delayed speech and language development, failure to thrive, hydronephrosis, omphalocele, premature birth, seizures, ventricular septal defect
395,586	chr16: 66,542,499–88,242,499	22	Likely pathogenic	Aplasia/Hypoplasia of the hallux, broad forehead, broad hallux, delayed closure of the anterior fontanelle, epicanthus, flat occiput, frontal bossing, high anterior hairline, intellectual disability, microcephaly, muscular hypotonia, proportionate short stature, supernumerary nipple
413,188	chr16: 85,817,324–87,841,184	2	Likely pathogenic	Unknown
285,653	chr16: 86,011,033–87,846,920	1.8	Pathogenic	Unknown
287,253	chr16: 86,761,351–87,972,139	1.2	Likely pathogenic	Intellectual disability, lymphedema
326,419	chr16: 86,804,086–87,887,066	1.1	Likely pathogenic	Abnormality of movement, microcephaly, seizure
401,267	chr16: 86,877,615–87,868,052	0.99	Likely pathogenic	Abnormality of prenatal development or birth, broad nasal tip, hyperextensibility of the finger joints, hypertelorism, intellectual disability, umbilical hernia
338,652	chr16: 86,910,262–89,623,832	2.7	Unknown	Acute lymphoblastic leukemia
268,339	chr16: 87,044,630–87,779,387	0.73	Unknown	Barrel-shaped chest, delayed speech and language development, feeding difficulties in infancy, intellectual disability, obesity
369,852	chr16: 87,054,643–87,410,554	0.36	Uncertain	Bilateral tonic–clonic seizure with focal onset, low-set ears, macrocephaly, neonatal hypotonia
400,063	chr16: 87,152,792–87,845,741	0.69	Likely pathogenic	Abnormality of the uterus, borneal ulceration, diabetes, distichiasis, intellectual disability, misalignment of teeth, predominantly lower limb lymphedema, ureter duplex, vesicoureteral reflux
289,169	chr16: 87,183,661–89,520,803	2.3	Uncertain	Abnormal facial shape, abnormality of the kidney, congenital thrombocytopenia, developmental delay, increased susceptibility to fractures, mild intrauterine growth retardation, multiple skeletal anomalies, ptosis
267,497	chr16: 87,213,968–87,504,677	0.29	Unknown	Unknown
321,856	chr16: 87,219,866–89,561,087	2.3	Likely pathogenic	Depressed nasal bridge, failure to thrive in infancy, moderate global developmental delay, prominent metopic ridge, rocker bottom foot, thick eyebrow, wide nose
280,733	chr16: 87,257,185–87,845,882	0.59	Unknown	2–3 toe syndactyly, behavioral abnormality, developmental delay, synophrys
360,400	chr16: 87,257,185–87,845,882	0.59	Likely pathogenic	Unknown
260,745	chr16: 87,319,450–88,669,353	1.4	Unknown	Depressed nasal bridge, hypotonia, hypertelorism, infra-orbital crease, prominent forehead, thoracic hypoplasia
255,327	chr16: 87,340,135–89,335,428	2	Unknown	Coarse facial features, intellectual disability, macrocephaly, obesity, proportionate short stature, short foot, short palm, synophrys
332,844	chr16: 87,381,358–87,713,863	0.33	Uncertain	Developmental delay
300,919	chr16: 87,432,206–89,199,829	1.8	Uncertain	Congenital horizontal nystagmus, high myopia, intellectual disability, joint hypermobility
263,364	chr16: 87,433,214–87,485,161	0.052	Unknown	Unknown
355,808	chr16: 87,439,917–87,753,353	0.31	Uncertain	Intellectual disability, obesity
411,602	chr16: 87,485,102–87,814,273	0.33	Uncertain	Intellectual disability
343,346	chr16: 87,504,276–87,670,757	0.17	Uncertain	Intellectual disability
339,346	chr16: 87,539,375–87,637,824	0.098	Unknown	Unknown
274,894	chr16: 87,539,375-87,845,741	0.31	Unknown	Developmental delay
277,919	chr16: 87,609,569–87,848,970	0.24	Unknown	Behavioral abnormality
289,590	chr16: 87,709,267–87,802,845	0.094	Uncertain	Behavioral abnormality, intellectual disability
288,635	chr16: 87,709,267–87,957,415	0.25	Uncertain	Developmental delay, tetralogy of Fallot

Based on the WES analyses of the trio (proband *versus* both parents), we did not find in the proband any de novo pathogenic or likely pathogenic variant located in the 1321 genes linked with monogenic forms of diabetes, obesity, kidney disorders, lymphedema, intellectual deficiency and/or distichiasis (Additional file 1: Table S1). However, we found one new heterozygous, likely pathogenic, missense variant in *WFS1* (NM_006005.3: c.424G > C/p.V142L). The heterozygous *WFS1* variants have previously been found to cause autosomal dominant diabetes [8]. This mutation that has never been reported before and that was not listed in the Genome Aggregation Database (gnomAD; v2.1.1), was inherited from her father who presented with type 2 diabetes. Furthermore, we found a likely pathogenic missense variant in *USP9X* (NM_001039590.2: c.3803A > G/p.Y1268C) involved in X-linked intellectual disability [9], which was inherited from her mother who had academic difficulties, related to dyscalculia and dyspraxia. One of the two healthy brothers of the patient did not carry neither *WFS1* p.V142L variant or *USP9X* p.Y1268C variant. In the proband, we did not identify any rare coding mutations in *FOXC2*.

## Discussion

We describe the case of a young female whose diabetes was difficult to associate with intellectual disability, polymalformative syndrome and lymphedema distichiasis syndrome (LDS).

On the one hand, initially, the young age of the patient, the presence of ketone bodies and the low C-peptide level at diagnosis, argued in favor of type 1 diabetes [10]. However, the absence of acidosis and of islets antibodies at diagnosis, the absence of anti-GAD, anti-IA2 and anti-ZNT8 antibodies at age 24 [11, 12], as well as the normal size of pancreas [13], suggested instead an atypical non-autoimmune diabetes. Furthermore, as the introduction of metformin in addition to insulin led to weight loss and a decrease in insulin needs, we hypothesized that insulin resistance was also involved in the patient’s metabolic phenotype. More recently, the unexpected effect of Liraglutide on the discontinuation of prandial insulin has been an additional argument in favor of atypical non-autoimmune diabetes [14–16].

On the other hand, we performed genetic investigations in front of a polymalformative syndrome and an intellectual disability.

Through our comprehensive genetic analyses, we identified a de novo 693-kb deletion in chr16q24.2. Intellectual disability, obesity, lymphedema and distichiasis have already been described in individuals carrying a pathogenic deletion at the same locus chr16q24.2 [17–19]. Interestingly, *FOXC2* that is a major gene linked with LDS (OMIM# 153400 [20]) was located close to the chr16q24.2 deletion (550 kb downstream). The specific function of *FOXC2* has not yet been fully explored; however, the encoded transcription factor plays an important role in the development and maintenance of venous and lymphatic valves and of mesenchymal tissues [21]. Considering that the activity of a transcription factor depends on its expression, we hypothesized that LDS might be the result of altered expression of *FOXC2*, triggered by the deletion of a long-range regulatory element in chr16q24.2 [7, 17, 22]. Indeed, a growing body of research suggests that deletions can exert a pathogenic effect through disruption of DNA structural elements such as TADs, and disruption of boundaries as a major mechanism [23]. TAD can regulate gene expression notably by the activation of an enhancer-promoter loop [24]. As the chr16q24.2 deletion encompassed two TADs, it might cause TAD disruption, and altered *FOXC2* expression leading to LDS [7, 23]. However, further studies are needed to demonstrate this assumption.

Furthermore, we identified a novel heterozygous, likely pathogenic, missense mutation in *WFS1* (p.V142L) inherited from the father with diabetes. Interestingly, we observed different presentations of diabetes in the proband and her father. Contrary to the proband, diabetes in the father was polygenic-like type 2 diabetes, as it was diagnosed in adulthood and was treated with metformin and DPP4 inhibitor without any insulin. Wolfram syndrome 1 caused by homozygous or compound heterozygous mutations in *WFS1* (encoding wolframin, a 100-kDa transmembrane glycoprotein localized in the secretory granules and endoplasmic reticulum [ER]). Heterozygous mutations in *WFS1* have been reported to be involved in less severe phenotypes including isolated adult-onset diabetes (OMIM #614296) [8]. A recent study has shown that GLP1-RA could be efficient for patients with diabetes and heterozygous, pathogenic *WFS1* variants [16]. The authors proposed that GLP1-RA might improve glucose regulation due to decreased ER stress leading to conservation of β-cell function and insulin secretion [16]. Here, we hypothesized that the likely pathogenic heterozygous variant in *WFS1* might possibly explain the patient's early-onset diabetes and the efficacy of GLP1-RA on prandial insulin interruption.

In addition, we found a likely pathogenic missense variant in *USP9X* (p.Y1268C) which was inherited from her mother who had dyscalculia and dyspraxia during her school career. *USP9X* encodes an enzyme that plays a key role in human neural development and the p.Y1268C mutation has been shown to be involved in X-linked intellectual disability [9]. Furthermore, *USP9X* is an X-chromosome gene that escapes X-inactivation [25]. Female patients with de novo pathogenic mutations present with mild to moderate intellectual disability (motor and language delay), and several other clinical features (including notably urogenital abnormality, hearing impairment, cleft palate or bifid uvula) [26]. The intellectual disability could therefore be due to the likely pathogenic missense variant in *USP9X* associated with the impact of the deletion.

The major limitation of our study is the fact that we did not analyze the functional effect of the deletion on *FOXC2* expression, and of the two variants in *WFS1* and *USP9X* on the activity of respective encoded proteins.

## Conclusions

In conclusion, we suggest that the patient’s complex syndrome might be due to several genetic events including a new likely pathogenic heterozygous *WFS1* variant, a likely pathogenic missense *USP9X* variant and a chr16q24.2 deletion possibly causing the dysregulation of *FOXC2* via TADs disruption (in addition to possibly other abnormalities due to the multi-gene deletion). This study demonstrates the relevance and usefulness of comprehensive genetic analyses in cases presenting with very complex phenotypes.

## Methods

### Patient

We collected clinical data of a female patient from pediatric and adult diabetology medical records. Family medical data have been also collected. In accordance with French laws, patient’s and her parents’ consent were obtained.

### DNA extraction

Genomic DNA samples from the proband and her two parents were extracted from peripheral blood using the QIAamp DNA Blood Midi kit (Qiagen, Valencia, CA, USA).

### Karyotyping

Conventional cytogenetic analysis was performed on peripheral blood lymphocytes from the patient using the 550-band including GTG (G-bands after trypsin and Giemsa) and RHG (R-bands by heating using Giemsa) banding for family members.

### DNA microarray

Array CGH was performed in the proband, using Agilent 180 k oligoarrays (SurePrint G3 Human CGH Microarray 4 × 180 k). Random primer labelling and hybridization were carried out with sex-matched reference DNA according to the manufacturer's recommendations and results were analyzed using cytogenomics (v3.0.6.6) software (Agilent Technologies) using ADM2 algorithm and a three-point filter.

CNV presence was confirmed in the proband and both parents by real-time PCR using primers targeting *JPH3* (that was included in the CNV). Primers for the *JPH3* was designed and tested using standard procedures (forward primer 5′-CCTGTGCGTCTGATCTGCT-3′, reverse primer 5′-GTCCTGCTGCTCCTTCTGAC-3′) on a LightCycler 480 Real-Time PCR System (Roche Diagnostics, Basel, Switzerland).

### Whole-exome sequencing (WES)

WES was performed in the proband and both parents. For this purpose, we used SeqCap EZ MedExome + Probes (Roche) or Human Core Exome Kit (Twist Bioscience), for Illumina sequencing (on the NovaSeq6000 system). Alignment of sequence reads to the human genome (GRCh38/hg38), variant calling and variant annotation were done as previously described [27]. For the proband and both parents, more than 98% of the target was covered with more than eight reads. Based on the clinical data of the patient (Fig. 1), we analyzed 1321 genes linked with monogenic diabetes, monogenic obesity, monogenic kidney disorders, lymphedema and/or intellectual deficiency (Additional file 1: Table S1). To assess the pathogenicity of the variants we used the standards and guidelines of the American College of Medical Genetics and Genomics (ACMG) [28]. For the moderate pathogenic criterion PM2 and the supporting pathogenic criterion PP2, we used gnomAD browser (v2.1.1). For the supporting pathogenic criterion PP3, we used PolyPhen-2 (HumDiv), and Mutation Taster [29, 30]. The variants were written according to the nomenclature of the Human Genome Variation Society (HGVS).

### TAD prediction

To integrate recent high-throughput technologies based on chromatin interaction data at the chr16q24.2 locus, we used the 3D Genome Browser (http://3dgenome.org), provided by the Bioinformatics and Genomics Program, Pennsylvania State University [31]. TADs were predicted from Hi-C analysis in Human fetal brain GZ [32].

## Supplementary Information

**Additional file 1: Supplementary Table 1**. Characteristics of the 1,321 genes that have been specifically analyzed in the WES data, according to the phenotypes of the patient.

## Data Availability

Please contact author for data requests.
