# Supplementary material for: Compound genetic etiology in a patient with a syndrome including diabetes, intellectual deficiency and distichiasis
Source: Orphanet J Rare Dis. 2022 Feb 28;17:86. doi: 10.1186/s13023-022-02248-2 (PMC8887189; doi:10.1186/s13023-022-02248-2)
Supplement: Supplementary file 1 — Additional file 1: Supplementary Table 1. Characteristics of the 1,321 genes that have been specifically analyzed in the WES data, according to the phenotypes of the patient. [file 13023_2022_2248_MOESM1_ESM.docx]

# Supplementary Table 1. Characteristics of the 1,321 genes that have been specifically analyzed in the WES data, according to the phenotypes of the patient

| Gene ID | Transcript | Diabetes | Obesity | Intellectual deficiency | Kidney disorders | Lymphoe-dema | Distichiasis |
| --- | --- | --- | --- | --- | --- | --- | --- |
| A2ML1 | NM_144670.5 |  |  | x |  |  |  |
| ABCC6 | NM_001171.5 |  |  |  | x |  |  |
| ABCC8 | NM_000352.4 | x |  |  |  |  |  |
| ABCC9 | NM_005691.3 |  |  | x |  |  |  |
| ABCD4 | NM_005050.3 |  |  |  | x |  |  |
| ABCG2 | NM_004827.2 |  |  |  | x |  |  |
| ACACB | NM_001093.3 |  | x |  |  |  |  |
| ACAD9 | NM_014049.4 |  |  | x |  |  |  |
| ACAN | NM_013227.3 |  |  | x |  |  |  |
| ACAT1 | NM_000019.3 |  |  | x |  |  |  |
| ACE | NM_000789.3 |  |  |  | x |  |  |
| ACP5 | NM_001111035.2 |  |  |  | x |  |  |
| ACSL4 | NM_004458.2 |  |  | x |  |  |  |
| ACTA2 | NM_001613.3 |  |  |  | x |  |  |
| ACTB | NM_001101.4 |  |  | x | x |  |  |
| ACTG1 | NM_001614.3 |  |  | x | x |  |  |
| ACTG2 | NM_001615.3 |  |  |  | x |  |  |
| ACTL6B | NM_016188.4 |  |  | x |  |  |  |
| ACTN4 | NM_004924.5 |  |  |  | x |  |  |
| ACVRL1 | NM_000020.2 |  |  |  | x |  |  |
| ACY1 | NM_000666.2 |  |  | x |  |  |  |
| ADA | NM_000022.3 |  |  | x |  |  |  |
| ADA2 | NM_001282225.1 |  |  |  | x |  |  |
| ADAMTS13 | NM_139025.4 |  |  |  | x |  |  |
| ADAMTS9 | NM_182920.1 |  |  |  | x |  |  |
| ADARB1 | NM_015833.3 |  |  | x |  |  |  |
| ADCY10 | NM_018417.5 |  |  |  | x |  |  |
| ADCY3 | NM_004036.4 |  | x |  |  |  |  |
| ADNP | NM_015339.4 |  |  | x |  |  |  |
| ADPRHL2 | NM_017825.2 |  |  | x |  |  |  |
| ADSL | NM_000026.3 |  |  | x |  |  |  |
| AFF2 | NM_002025.3 |  | x | x |  |  |  |
| AFF4 | NM_014423.3 |  | x | x | x |  |  |
| AGA | NM_000027.3 |  |  | x |  |  |  |
| AGO1 | NM_012199.4 |  |  | x |  |  |  |
| AGPAT2 | NM_006412.3 |  |  |  | x |  |  |
| AGT | NM_000029.3 |  |  |  | x |  |  |
| AGTR1 | NM_031850.3 |  |  |  | x |  |  |
| AGXT | NM_000030.2 |  |  |  | x |  |  |
| AHDC1 | NM_001029882.3 |  |  | x |  |  |  |
| AHI1 | NM_017651.4 |  |  | x | x |  |  |
| ALDH18A1 | NM_002860.3 |  |  | x |  |  |  |
| ALDH3A2 | NM_000382.2 |  |  | x |  |  |  |
| ALDH5A1 | NM_001080.3 |  |  | x |  |  |  |
| ALDH7A1 | NM_001182.4 |  |  | x |  |  |  |
| ALDOB | NM_000035.3 | x |  | x |  |  |  |
| ALG1 | NM_019109.4 |  |  | x |  |  |  |
| ALG12 | NM_024105.3 |  |  | x |  |  |  |
| ALG13 | NM_001099922.2 |  |  | x |  |  |  |
| ALG3 | NM_005787.5 |  |  | x |  |  |  |
| ALG6 | NM_013339.3 |  |  | x |  |  |  |
| ALG8 | NM_024079.4 |  |  |  | x |  |  |
| ALG9 | NM_024740.2 |  |  |  | x |  |  |
| ALKBH8 | NM_001301010.1 |  |  | x |  |  |  |
| ALMS1 | NM_015120.4 |  | x | x | x |  |  |
| ALPL | NM_000478.5 |  |  |  | x |  |  |
| AMER1 | NM_152424.3 |  |  |  | x |  |  |
| AMN | NM_030943.3 |  |  |  | x |  |  |
| AMT | NM_000481.3 |  |  | x |  |  |  |
| ANK2 | NM_001148.5 |  |  | x |  |  |  |
| ANK3 | NM_020987.4 |  |  | x |  |  |  |
| ANKH | NM_054027.5 |  |  | x |  |  |  |
| ANKRD1 | NM_014391.2 |  |  |  | x |  |  |
| ANKRD11 | NM_013275.5 |  |  | x |  |  |  |
| ANKS1B | NM_152788.4 |  |  | x |  |  |  |
| ANKS6 | NM_173551.4 |  |  |  | x |  |  |
| ANLN | NM_018685.4 |  |  |  | x |  |  |
| ANOS1 | NM_000216.3 |  | x | x | x |  |  |
| AP1S2 | NM_003916.4 |  |  | x |  |  |  |
| AP2M1 | NM_004068.3 |  |  | x |  |  |  |
| AP2S1 | NM_004069.4 |  |  |  | x |  |  |
| AP4B1 | NM_006594.4 |  |  | x |  |  |  |
| AP4E1 | NM_007347.4 |  |  | x |  |  |  |
| AP4M1 | NM_004722.3 |  |  | x |  |  |  |
| AP4S1 | NM_007077.4 |  |  | x |  |  |  |
| APC2 | NM_005883.2 |  |  | x | x |  |  |
| APOA1 | NM_000039.2 |  |  |  | x |  |  |
| APPL1 | NM_012096.2 | x |  |  |  |  |  |
| APRT | NM_000485.2 |  |  |  | x |  |  |
| AQP1 | NM_198098.3 |  |  |  | x |  |  |
| AQP2 | NM_000486.5 |  |  |  | x |  |  |
| ARFGEF2 | NM_006420.2 |  |  | x |  |  |  |
| ARHGDIA | NM_001185077.2 |  |  |  | x |  |  |
| ARHGEF6 | NM_004840.2 |  |  | x |  |  |  |
| ARHGEF9 | NM_015185.2 |  |  | x |  |  |  |
| ARID1A | NM_006015.5 |  |  | x | x |  |  |
| ARID1B | NM_020732.3 |  |  | x | x |  |  |
| ARIH1 | NM_005744.3 |  |  | x |  |  |  |
| ARL13B | NM_182896.2 |  |  |  | x |  |  |
| ARL6 | NM_177976.3 |  | x | x | x |  |  |
| ARMC5 | NM_001105247.1 |  |  |  | x |  |  |
| ARNT2 | NM_014862.3 |  |  |  | x |  |  |
| ARSA | NM_000487.5 |  |  | x |  |  |  |
| ARX | NM_139058.2 |  |  | x | x |  |  |
| ASAH1 | NM_177924.4 |  |  | x |  |  |  |
| ASH1L | NM_018489.2 |  |  | x |  |  |  |
| ASL | NM_000048.3 |  |  | x |  |  |  |
| ASPA | NM_000049.2 |  |  | x |  |  |  |
| ASPM | NM_018136.4 |  |  | x |  |  |  |
| ASS1 | NM_000050.4 |  |  | x |  |  |  |
| ASXL1 | NM_015338.5 |  |  | x | x |  |  |
| ASXL3 | NM_030632.2 |  |  | x |  |  |  |
| ATIC | NM_004044.6 |  |  | x |  |  |  |
| ATP1A1 | NM_000701.7 |  |  | x |  |  |  |
| ATP6AP2 | NM_005765.2 |  |  | x |  |  |  |
| ATP6V0A4 | NM_020632.2 |  |  |  | x |  |  |
| ATP6V1B1 | NM_001692.3 |  |  |  | x |  |  |
| ATP6V1C2 | NM_001039362.1 |  |  |  | x |  |  |
| ATP7A | NM_000052.6 |  |  | x | x |  |  |
| ATP7B | NM_000053.3 |  |  | x | x |  |  |
| ATR | NM_001184.3 |  |  | x |  |  |  |
| ATRX | NM_000489.4 |  | x | x | x |  |  |
| ATXN10 | NM_013236.3 |  |  |  | x |  |  |
| AUH | NM_001698.2 |  |  |  | x |  |  |
| AUTS2 | NM_015570.3 |  |  | x |  |  |  |
| AVP | NM_000490.4 |  |  |  | x |  |  |
| AVPR2 | NM_000054.4 |  |  |  | x |  |  |
| B2M | NM_004048.2 |  |  |  | x |  |  |
| B3GLCT | NM_194318.3 |  |  | x | x |  |  |
| B4GAT1 | NM_006876.2 |  |  |  | x |  |  |
| B9D1 | NM_015681.4 |  |  |  | x |  |  |
| B9D2 | NM_030578.3 |  |  |  | x |  |  |
| BBIP1 | NM_001195306.1 |  | x | x | x |  |  |
| BBS1 | NM_024649.4 |  | x | x | x |  |  |
| BBS10 | NM_024685.3 |  | x | x | x |  |  |
| BBS12 | NM_152618.2 |  | x | x | x |  |  |
| BBS2 | NM_031885.3 |  | x | x | x |  |  |
| BBS4 | NM_033028.4 |  | x | x | x |  |  |
| BBS5 | NM_152384.2 |  | x | x | x |  |  |
| BBS7 | NM_176824.2 |  | x | x | x |  |  |
| BBS9 | NM_198428.2 |  | x | x | x |  |  |
| BCKDHA | NM_000709.3 |  |  | x |  |  |  |
| BCKDHB | NM_183050.3 |  |  | x |  |  |  |
| BCKDK | NM_005881.3 |  |  | x |  |  |  |
| BCL11A | NM_022893.3 |  |  | x |  |  |  |
| BCOR | NM_017745.5 |  |  | x | x |  |  |
| BCR | NM_004327.3 |  |  |  | x |  |  |
| BCS1L | NM_004328.4 |  |  |  | x |  |  |
| BDNF | NM_170735.5 |  | x |  |  |  |  |
| BLK | NM_001715.2 | x |  |  |  |  |  |
| BMP4 | NM_001202.5 |  |  |  | x |  |  |
| BMP7 | NM_001719.2 |  |  |  | x |  |  |
| BMPER | NM_133468.4 |  |  |  | x |  |  |
| BRAF | NM_004333.5 |  |  | x | x |  |  |
| BRD3 | NM_007371.3 |  |  | x |  |  |  |
| BRIP1 | NM_032043.2 |  |  |  | x |  |  |
| BRPF1 | NM_001003694.1 |  |  | x |  |  |  |
| BRWD1 | NM_018963.4 |  |  | x |  |  |  |
| BRWD3 | NM_153252.4 |  | x | x |  |  |  |
| BSCL2 | NM_032667.6 |  |  |  | x |  |  |
| BSND | NM_057176.2 |  |  |  | x |  |  |
| BTD | NM_001281723.2 |  |  | x |  |  |  |
| BTF3 | NM_001037637.1 |  |  | x |  |  |  |
| BUB1B | NM_001211.5 |  |  |  | x |  |  |
| C12orf57 | NM_138425.3 |  |  | x |  |  |  |
| C1QA | NM_015991.3 |  |  |  | x |  |  |
| C1QB | NM_000491.4 |  |  |  | x |  |  |
| C1QC | NM_172369.4 |  |  |  | x |  |  |
| C2 | NM_000063.5 |  |  |  | x |  |  |
| C2CD3 | NM_015531.5 |  |  |  | x |  |  |
| C3 | NM_000064.3 |  |  |  | x |  |  |
| C4A | NM_007293.2 |  |  |  | x |  |  |
| CA2 | NM_000067.2 |  |  |  | x |  |  |
| CA8 | NM_004056.5 |  |  | x |  |  |  |
| CACNA1C | NM_000719.6 |  |  | x |  |  |  |
| CACNA1D | NM_000720.3 | x |  | x |  |  |  |
| CACNA1E | NM_000721.3 |  |  | x |  |  |  |
| CACNA1F | NM_005183.3 |  |  | x |  |  |  |
| CACNA1G | NM_018896.4 |  |  | x |  |  |  |
| CACNA1S | NM_000069.2 |  |  |  | x |  |  |
| CACNG2 | NM_006078.4 |  |  | x |  |  |  |
| CAD | NM_004341.4 |  |  |  | x |  |  |
| CALCR | NM_001742.3 |  |  |  | x |  |  |
| CAMK2A | NM_015981.3 |  |  | x |  |  |  |
| CAMTA1 | NM_015215.3 |  |  | x |  |  |  |
| CASK | NM_003688.3 |  |  | x |  |  |  |
| CASP10 | NM_032977.3 |  |  |  | x |  |  |
| CASR | NM_000388.3 |  |  | x | x |  |  |
| CBL | NM_005188.3 |  |  | x |  |  |  |
| CBS | NM_000071.2 |  |  | x |  |  |  |
| CBWD1 | NM_018491.3 |  |  |  | x |  |  |
| CC2D1A | NM_017721.4 |  |  | x |  |  |  |
| CC2D2A | NM_001080522.2 |  |  |  | x |  |  |
| CCBE1 | NM_133459.3 |  |  |  | x | x |  |
| CCDC22 | NM_014008.4 |  |  | x | x |  |  |
| CCDC47 | NM_020198.2 |  |  | x |  |  |  |
| CCNQ | NM_152274.4 |  |  |  | x |  |  |
| CD151 | NM_004357.4 |  |  |  | x |  |  |
| CD19 | NM_001770.5 |  |  |  | x |  |  |
| CD81 | NM_004356.3 |  |  |  | x |  |  |
| CD96 | NM_198196.2 |  |  |  | x |  |  |
| CDC5L | NM_001253.3 |  |  |  | x |  |  |
| CDC73 | NM_024529.4 |  |  |  | x |  |  |
| CDH10 | NM_006727.4 |  |  | x |  |  |  |
| CDH15 | NM_004933.2 |  |  | x |  |  |  |
| CDH2 | NM_001792.4 |  |  | x |  |  |  |
| CDH9 | NM_016279.3 |  |  | x |  |  |  |
| CDK13 | NM_003718.4 |  |  | x |  |  |  |
| CDK19 | NM_001300960.1 |  |  | x |  |  |  |
| CDK5RAP2 | NM_018249.5 |  |  | x |  |  |  |
| CDK6 | NM_001259.7 |  |  | x |  |  |  |
| CDK8 | NM_001260.2 |  |  | x |  |  |  |
| CDKL5 | NM_003159.2 |  | x | x |  |  |  |
| CDKN1B | NM_004064.4 |  |  |  | x |  |  |
| CDKN1C | NM_000076.2 |  |  |  | x |  |  |
| CEL | NM_001807.6 | x |  |  |  |  |  |
| CELA2A | NM_033440.2 | x | x |  |  |  |  |
| CELSR1 | NM_014246.1 |  |  |  |  | x |  |
| CENPF | NM_016343.3 |  |  |  | x |  |  |
| CENPJ | NM_018451.4 |  |  | x |  |  |  |
| CEP104 | NM_014704.3 |  |  |  | x |  |  |
| CEP120 | NM_153223.3 |  |  |  | x |  |  |
| CEP152 | NM_014985.3 |  |  | x |  |  |  |
| CEP164 | NM_014956.4 |  |  |  | x |  |  |
| CEP19 | NM_032898.4 |  | x | x |  |  |  |
| CEP290 | NM_025114.3 |  | x | x | x |  |  |
| CEP41 | NM_018718.2 |  |  |  | x |  |  |
| CEP83 | NM_016122.2 |  |  |  | x |  |  |
| CERT1 | NM_001130105.1 |  |  | x |  |  |  |
| CFB | NM_001710.5 |  |  |  | x |  |  |
| CFH | NM_000186.3 |  |  |  | x |  |  |
| CFHR1 | NM_002113.2 |  |  |  | x |  |  |
| CFHR3 | NM_021023.5 |  |  |  | x |  |  |
| CFHR5 | NM_030787.3 |  |  |  | x |  |  |
| CFI | NM_000204.4 |  |  |  | x |  |  |
| CFTR | NM_000492.3 |  |  |  | x |  |  |
| CHAMP1 | NM_001164144.2 |  |  | x |  |  |  |
| CHD1L | NM_004284.5 |  |  |  | x |  |  |
| CHD2 | NM_001271.3 |  | x | x |  |  |  |
| CHD4 | NM_001273.3 |  |  | x |  |  |  |
| CHD5 | NM_015557.2 |  |  | x |  |  |  |
| CHD7 | NM_017780.3 |  | x | x | x |  |  |
| CHD8 | NM_001170629.1 |  |  | x |  |  |  |
| CHRM3 | NM_000740.3 |  |  |  | x |  |  |
| CHRNA2 | NM_000742.3 |  |  | x |  |  |  |
| CHRNA3 | NM_000743.4 |  |  |  | x |  |  |
| CHRNA4 | NM_000744.6 |  |  | x |  |  |  |
| CHRNA7 | NM_000746.5 |  |  | x |  |  |  |
| CHRNB2 | NM_000748.2 |  |  | x |  |  |  |
| CHST14 | NM_130468.3 |  |  |  | x |  |  |
| CISD2 | NM_001008388.4 | x |  |  | x |  |  |
| CLCN2 | NM_004366.5 |  |  | x |  |  |  |
| CLCN4 | NM_001830.3 |  |  | x |  |  |  |
| CLCN5 | NM_000084.4 |  |  |  | x |  |  |
| CLCN6 | NM_001286.3 |  |  | x |  |  |  |
| CLCNKA | NM_004070.3 |  |  |  | x |  |  |
| CLCNKB | NM_000085.4 |  |  |  | x |  |  |
| CLDN10 | NM_006984.4 |  |  |  | x |  |  |
| CLDN14 | NM_144492.2 |  |  |  | x |  |  |
| CLDN16 | NM_006580.3 |  |  |  | x |  |  |
| CLDN19 | NM_148960.2 |  |  |  | x |  |  |
| CLDN2 | NM_020384.3 |  |  |  | x |  |  |
| CLIC2 | NM_001289.5 |  |  | x |  |  |  |
| CLN3 | NM_001042432.1 |  |  | x |  |  |  |
| CLN5 | NM_006493.4 |  |  | x |  |  |  |
| CLN6 | NM_017882.2 |  |  | x |  |  |  |
| CLN8 | NM_018941.3 |  |  | x |  |  |  |
| CLTC | NM_001288653.1 |  |  | x |  |  |  |
| CNKSR2 | NM_014927.4 |  |  | x |  |  |  |
| CNNM2 | NM_017649.4 |  |  |  | x |  |  |
| CNOT1 | NM_001265612.1 | x |  | x |  |  |  |
| CNOT3 | NM_014516.3 |  |  | x |  |  |  |
| CNTN4 | NM_175607.2 |  |  | x |  |  |  |
| CNTNAP2 | NM_014141.5 |  |  | x |  |  |  |
| COA8 | NM_001370595.2 |  |  |  | x |  |  |
| COL18A1 | NM_130445.3 |  |  |  | x |  |  |
| COL4A1 | NM_001845.5 |  |  |  | x |  |  |
| COL4A3 | NM_000091.4 |  |  |  | x |  |  |
| COL4A4 | NM_000092.4 |  |  |  | x |  |  |
| COL4A5 | NM_000495.4 |  |  |  | x |  |  |
| COL5A1 | NM_000093.4 |  |  |  | x |  |  |
| COPA | NM_004371.3 |  |  |  | x |  |  |
| COQ2 | NM_015697.8 |  |  |  | x |  |  |
| COQ6 | NM_182476.2 |  |  |  | x |  |  |
| COQ7 | NM_016138.4 |  |  |  | x |  |  |
| COQ8A | NM_020247.4 |  |  | x |  |  |  |
| COQ8B | NM_024876.3 |  |  |  | x |  |  |
| COQ9 | NM_020312.3 |  |  |  | x |  |  |
| COX10 | NM_001303.3 |  |  |  | x |  |  |
| COX14 | NM_032901.3 |  |  |  | x |  |  |
| COX20 | NM_198076.5 |  |  |  | x |  |  |
| COX6B1 | NM_001863.4 |  |  |  | x |  |  |
| COX7B | NM_001866.2 |  |  |  | x |  |  |
| COX8A | NM_004074.2 |  |  |  | x |  |  |
| CPA6 | NM_020361.4 |  |  | x |  |  |  |
| CPE | NM_001873.3 |  | x | x |  |  |  |
| CPLANE1 | NM_023073.3 |  |  |  | x |  |  |
| CPS1 | NM_001875.4 |  |  | x |  |  |  |
| CPT1A | NM_001876.3 |  |  |  | x |  |  |
| CPT2 | NM_000098.2 |  |  |  | x |  |  |
| CRB2 | NM_173689.6 |  |  |  | x |  |  |
| CRBN | NM_016302.3 |  |  | x |  |  |  |
| CREBBP | NM_004380.2 |  | x | x | x |  |  |
| CRTAP | NM_006371.4 |  |  |  | x |  |  |
| CSDE1 | NM_001130523.2 |  |  | x |  |  |  |
| CSNK2A1 | NM_001895.3 |  |  | x |  |  |  |
| CSPP1 | NM_024790.6 |  |  |  | x |  |  |
| CSTB | NM_000100.3 |  |  | x |  |  |  |
| CTC1 | NM_025099.5 |  |  |  | x |  |  |
| CTCF | NM_006565.3 |  |  | x |  |  |  |
| CTNNB1 | NM_001904.3 |  |  | x |  |  |  |
| CTNS | NM_004937.2 |  |  |  | x |  |  |
| CTRC | NM_007272.2 |  |  |  | x |  |  |
| CTSF | NM_003793.3 |  |  | x |  |  |  |
| CTU2 | NM_001012759.2 |  |  |  | x |  |  |
| CUBN | NM_001081.3 |  |  |  | x |  |  |
| CUL3 | NM_003590.4 |  |  | x | x |  |  |
| CUL4B | NM_003588.3 |  | x | x |  |  |  |
| CUX2 | NM_015267.3 |  |  | x |  |  |  |
| CYFIP1 | NM_014608.5 |  |  | x |  |  |  |
| CYP11A1 | NM_000781.2 |  |  |  | x |  |  |
| CYP11B1 | NM_000497.3 |  |  |  | x |  |  |
| CYP17A1 | NM_000102.3 |  |  |  | x |  |  |
| CYP21A2 | NM_000500.7 |  |  |  | x |  |  |
| CYP24A1 | NM_000782.4 |  |  |  | x |  |  |
| CYP27A1 | NM_000784.3 |  |  | x |  |  |  |
| CYP27B1 | NM_000785.3 |  |  |  | x |  |  |
| CYP2R1 | NM_024514.4 |  |  |  | x |  |  |
| CYP2U1 | NM_183075.2 |  |  | x |  |  |  |
| D2HGDH | NM_152783.4 |  |  | x |  |  |  |
| DAAM2 | NM_015345.3 |  |  |  | x |  |  |
| DBT | NM_001918.3 |  |  | x |  |  |  |
| DCDC2 | NM_016356.4 |  |  |  | x |  |  |
| DCHS1 | NM_003737.3 |  |  |  | x |  |  |
| DCX | NM_178153.2 |  |  | x |  |  |  |
| DDHD2 | NM_015214.2 |  |  | x |  |  |  |
| DDX3X | NM_001193416.2 |  |  | x |  |  |  |
| DDX59 | NM_001031725.5 |  |  |  | x |  |  |
| DDX6 | NM_004397.5 |  |  | x |  |  |  |
| DEAF1 | NM_021008.3 |  | x | x |  |  |  |
| DEPDC5 | NM_001242896.1 |  |  | x |  |  |  |
| DGKD | NM_152879.2 |  |  |  | x |  |  |
| DGKE | NM_003647.2 |  |  |  | x |  |  |
| DGKH | NM_152910.5 |  |  |  | x |  |  |
| DGUOK | NM_080916.2 | x |  |  |  |  |  |
| DHCR24 | NM_014762.3 |  |  | x |  |  |  |
| DHCR7 | NM_001360.2 |  |  | x | x |  |  |
| DIP2B | NM_173602.2 |  |  | x |  |  |  |
| DIS3L2 | NM_152383.4 |  |  |  | x |  |  |
| DISC1 | NM_018662.2 |  |  | x |  |  |  |
| DKC1 | NM_001363.4 |  |  | x | x |  |  |
| DLG2 | NM_001142699.1 |  |  | x |  |  |  |
| DLG3 | NM_021120.3 |  |  | x | x |  |  |
| DLL1 | NM_005618.3 |  |  | x |  |  |  |
| DLL3 | NM_016941.3 |  |  |  | x |  |  |
| DLL4 | NM_019074.3 |  |  |  | x |  |  |
| DLX4 | NM_138281.2 |  |  |  | x |  |  |
| DMD | NM_004006.2 |  |  | x |  |  |  |
| DMP1 | NM_004407.3 |  |  |  | x |  |  |
| DNA2 | NM_001080449.2 |  |  |  | x |  |  |
| DNAAF1 | NM_178452.5 |  |  |  | x |  |  |
| DNAJB11 | NM_016306.5 |  |  |  | x |  |  |
| DNAJC3 | NM_006260.4 | x |  |  |  |  |  |
| DNASE1L3 | NM_004944.3 |  |  |  | x |  |  |
| DNM1 | NM_004408.3 |  |  | x |  |  |  |
| DNM1L | NM_012062.4 |  |  | x |  |  |  |
| DNM3 | NM_015569.4 |  |  | x |  |  |  |
| DNMT3A | NM_175629.2 |  |  | x |  |  |  |
| DNMT3B | NM_006892.3 |  |  | x | x |  |  |
| DOCK6 | NM_020812.3 |  |  | x |  |  |  |
| DPAGT1 | NM_001382.3 |  |  | x |  |  |  |
| DPH1 | NM_001383.4 |  |  |  | x |  |  |
| DPM1 | NM_003859.2 |  |  | x |  |  |  |
| DPP6 | NM_001936.4 |  |  | x |  |  |  |
| DPYD | NM_000110.3 |  |  | x |  |  |  |
| DSCAM | NM_001389.4 |  |  | x |  |  |  |
| DSTYK | NM_015375.2 |  |  |  | x |  |  |
| DYNC1H1 | NM_001376.4 |  |  | x |  |  |  |
| DYNC1I2 | NM_001378.2 |  |  | x |  |  |  |
| DYNC2H1 | NM_001080463.1 |  |  |  | x |  |  |
| DYRK1A | NM_001396.4 |  |  | x | x |  |  |
| DYRK1B | NM_004714.2 | x | x |  |  |  |  |
| EBF3 | NM_001005463.2 |  |  | x |  |  |  |
| EBP | NM_006579.2 |  |  |  | x |  |  |
| EDNRA | NM_001957.3 |  |  |  | x |  |  |
| EEF1A2 | NM_001958.3 |  |  | x |  |  |  |
| EFEMP2 | NM_016938.4 |  |  |  | x |  |  |
| EFTUD2 | NM_004247.3 |  |  | x |  |  |  |
| EGF | NM_001963.5 |  |  |  | x |  |  |
| EHHADH | NM_001966.3 |  |  |  | x |  |  |
| EHMT1 | NM_024757.4 |  | x | x |  |  |  |
| EIF2AK3 | NM_004836.6 | x |  |  | x |  |  |
| EIF2B1 | NM_001414.3 | x |  |  |  |  |  |
| EIF2B4 | NM_015636.3 |  |  |  | x |  |  |
| EIF2S3 | NM_001415.3 |  | x | x |  |  |  |
| EIF4E | NM_001130679.2 |  |  | x |  |  |  |
| ELP1 | NM_003640.4 |  |  |  | x |  |  |
| ELP2 | NM_001242875.2 |  |  | x |  |  |  |
| EMC1 | NM_015047.2 |  |  | x |  |  |  |
| EMP2 | NM_001424.5 |  |  |  | x |  |  |
| EN2 | NM_001427.3 |  |  | x |  |  |  |
| ENG | NM_000118.3 |  |  |  | x |  |  |
| ENPP1 | NM_006208.2 |  |  |  | x |  |  |
| EP300 | NM_001429.3 |  | x | x |  |  |  |
| EPB41L1 | NM_012156.2 |  |  | x |  |  |  |
| EPG5 | NM_020964.2 |  |  |  | x |  |  |
| EPHA2 | NM_004431.4 |  |  |  | x |  |  |
| EPM2A | NM_005670.3 |  |  | x |  |  |  |
| ERBB3 | NM_001982.3 |  |  |  | x |  |  |
| ERCC4 | NM_005236.2 |  |  |  | x |  |  |
| ERCC6 | NM_000124.3 |  |  |  | x |  |  |
| ERCC8 | NM_000082.3 |  |  |  | x |  |  |
| ERLIN2 | NM_007175.6 |  |  | x |  |  |  |
| ESCO2 | NM_001017420.2 |  |  |  | x |  |  |
| ETFA | NM_000126.3 |  |  |  | x |  |  |
| ETFB | NM_001985.2 |  |  |  | x |  |  |
| ETFDH | NM_004453.3 |  |  |  | x |  |  |
| ETV4 | NM_001986.2 |  |  |  | x |  |  |
| EVC | NM_153717.2 |  |  |  | x |  |  |
| EVC2 | NM_147127.4 |  |  |  | x |  |  |
| EYA1 | NM_000503.5 |  |  |  | x |  |  |
| F2 | NM_000506.4 |  |  |  | x |  |  |
| FAH | NM_000137.2 |  |  |  | x |  |  |
| FAM20A | NM_017565.3 |  |  |  | x |  |  |
| FAM20C | NM_020223.3 |  |  |  | x |  |  |
| FAN1 | NM_014967.4 |  |  |  | x |  |  |
| FANCA | NM_000135.3 |  |  |  | x |  |  |
| FANCB | NM_001018113.2 |  |  |  | x |  |  |
| FANCC | NM_000136.2 |  |  |  | x |  |  |
| FANCD2 | NM_033084.4 |  |  |  | x |  |  |
| FANCE | NM_021922.2 |  |  |  | x |  |  |
| FANCF | NM_022725.3 |  |  |  | x |  |  |
| FANCG | NM_004629.1 |  |  |  | x |  |  |
| FANCI | NM_001113378.1 |  |  |  | x |  |  |
| FANCL | NM_018062.3 |  |  |  | x |  |  |
| FANCM | NM_020937.3 |  |  |  | x |  |  |
| FASTKD2 | NM_014929.3 |  |  |  | x |  |  |
| FAT4 | NM_024582.4 |  |  |  | x |  |  |
| FBLN5 | NM_006329.3 |  |  |  | x |  |  |
| FBN3 | NM_032447.4 |  | x | x | x |  |  |
| FBXL4 | NM_012160.4 |  |  |  | x |  |  |
| FBXO11 | NM_001190274.1 |  |  | x |  |  |  |
| FBXW11 | NM_012300.2 |  |  | x |  |  |  |
| FGA | NM_021871.3 |  |  |  | x |  |  |
| FGD1 | NM_004463.2 |  |  | x |  |  |  |
| FGF10 | NM_004465.1 |  |  |  | x |  |  |
| FGF20 | NM_019851.2 |  |  |  | x |  |  |
| FGF23 | NM_020638.2 |  |  |  | x |  |  |
| FGFR1 | NM_023110.2 |  | x | x | x |  |  |
| FGFR2 | NM_000141.4 |  |  |  | x |  |  |
| FGFR3 | NM_000142.4 |  |  |  | x |  |  |
| FKBP14 | NM_017946.3 |  |  |  | x |  |  |
| FLCN | NM_144997.6 |  |  |  | x |  |  |
| FLNA | NM_001456.3 |  |  | x | x |  |  |
| FLNB | NM_001457.3 |  |  |  | x |  |  |
| FLT4 | NM_182925.4 |  |  |  | x | x |  |
| FMR1 | NM_002024.5 |  | x | x |  |  |  |
| FN1 | NM_212482.2 |  |  |  | x |  |  |
| FOLR1 | NM_016725.2 |  |  | x |  |  |  |
| FOXA2 | NM_021784.4 | x |  |  |  |  |  |
| FOXC1 | NM_001453.2 |  |  |  | x |  |  |
| FOXC2 | NM_005251.2 |  |  |  | x | x | x |
| FOXF1 | NM_001451.2 |  |  |  | x |  |  |
| FOXG1 | NM_005249.4 |  |  | x |  |  |  |
| FOXP1 | NM_032682.5 |  |  | x |  |  |  |
| FOXP2 | NM_014491.3 |  |  | x |  |  |  |
| FOXP3 | NM_014009.3 | x |  |  |  |  |  |
| FRAS1 | NM_025074.6 |  |  |  | x |  |  |
| FREM1 | NM_144966.5 |  |  |  | x |  |  |
| FREM2 | NM_207361.5 |  |  |  | x |  |  |
| FRMPD4 | NM_014728.3 |  |  | x |  |  |  |
| FTCD | NM_006657.2 |  |  | x |  |  |  |
| FTSJ1 | NM_012280.3 |  |  | x |  |  |  |
| FUCA1 | NM_000147.4 |  |  | x |  |  |  |
| FUZ | NM_025129.4 |  |  |  | x |  |  |
| FXYD2 | NM_001680.4 |  |  |  | x |  |  |
| G6PC | NM_000151.3 |  |  |  | x |  |  |
| GABBR2 | NM_005458.7 |  |  | x |  |  |  |
| GABRA1 | NM_000806.5 |  |  | x |  |  |  |
| GABRB2 | NM_021911.2 |  |  | x |  |  |  |
| GABRB3 | NM_000814.5 |  |  | x |  |  |  |
| GABRD | NM_000815.4 |  |  | x |  |  |  |
| GABRG2 | NM_000816.3 |  |  | x |  |  |  |
| GALNT3 | NM_004482.3 |  |  |  | x |  |  |
| GALT | NM_000155.3 | x |  | x | x |  |  |
| GAMT | NM_000156.5 |  |  | x |  |  |  |
| GANAB | NM_198335.3 |  |  |  | x |  |  |
| GAP43 | NM_001130064.1 |  |  | x |  |  |  |
| GATA2 | NM_032638.4 |  |  |  |  | x |  |
| GATA3 | NM_001002295.1 |  |  |  | x |  |  |
| GATA4 | NM_002052.4 | x |  |  |  |  |  |
| GATA6 | NM_005257.5 | x |  |  | x |  |  |
| GATAD2B | NM_020699.3 |  |  | x |  |  |  |
| GATM | NM_001482.2 |  |  | x |  |  |  |
| GBA | NM_001005741.2 |  |  | x | x |  |  |
| GCDH | NM_000159.3 |  |  | x | x |  |  |
| GCH1 | NM_000161.2 |  |  | x |  |  |  |
| GCK | NM_000162.4 | x |  |  |  |  |  |
| GCM2 | NM_004752.3 |  |  |  | x |  |  |
| GCSH | NM_004483.4 |  |  | x |  |  |  |
| GDF15 | NM_004864.3 | x |  |  |  |  |  |
| GDF2 | NM_016204.3 |  |  |  | x |  |  |
| GDI1 | NM_001493.2 |  |  | x |  |  |  |
| GDNF | NM_000514.3 |  |  |  | x |  |  |
| GFPT2 | NM_005110.3 |  |  | x |  |  |  |
| GFRA1 | NM_005264.5 |  |  |  | x |  |  |
| GHR | NM_000163.4 |  | x | x |  |  |  |
| GIPC1 | NM_202470.2 |  |  |  | x |  |  |
| GJC2 | NM_020435.3 |  |  |  |  | x |  |
| GK | NM_000167.5 |  |  | x |  |  |  |
| GLA | NM_000169.2 |  |  |  | x |  |  |
| GLB1 | NM_000404.3 |  |  |  | x |  |  |
| GLDC | NM_000170.2 |  |  | x |  |  |  |
| GLI3 | NM_000168.5 |  |  |  | x |  |  |
| GLIS2 | NM_032575.2 |  |  |  | x |  |  |
| GLIS3 | NM_152629.3 | x |  |  | x |  |  |
| GLUD1 | NM_005271.4 | x |  |  |  |  |  |
| GNA11 | NM_002067.4 |  |  |  | x |  |  |
| GNAI1 | NM_002069.5 |  |  | x |  |  |  |
| GNAO1 | NM_020988.2 |  |  | x |  |  |  |
| GNAS | NM_000516.5 |  | x |  | x |  |  |
| GNB1 | NM_002074.4 |  |  |  | x |  |  |
| GOSR2 | NM_004287.4 |  |  | x |  |  |  |
| GPC3 | NM_004484.3 |  |  | x | x |  |  |
| GPRASP1 | NM_001099411.1 |  |  | x |  |  |  |
| GREB1L | NM_001142966.2 |  |  |  | x |  |  |
| GREM1 | NM_013372.6 |  |  |  | x |  |  |
| GRHPR | NM_012203.1 |  |  |  | x |  |  |
| GRIA3 | NM_000828.4 |  |  | x |  |  |  |
| GRIA4 | NM_000829.3 |  |  | x |  |  |  |
| GRID2 | NM_001510.3 |  |  | x |  |  |  |
| GRIK2 | NM_021956.4 |  |  | x |  |  |  |
| GRIN1 | NM_007327.3 |  |  | x |  |  |  |
| GRIN2A | NM_000833.4 |  |  | x |  |  |  |
| GRIN2B | NM_000834.4 |  |  | x |  |  |  |
| GRIP1 | NM_021150.3 |  |  | x | x |  |  |
| GSN | NM_000177.4 |  |  |  | x |  |  |
| GUSB | NM_000181.3 |  |  | x |  |  |  |
| H3-3A | NM_002107.4 |  |  | x |  |  |  |
| H3-3B | NM_005324.4 |  |  | x |  |  |  |
| HADH | NM_005327.4 | x |  |  |  |  |  |
| HADHA | NM_000182.4 | x |  |  |  |  |  |
| HADHB | NM_000183.2 | x |  |  |  |  |  |
| HAL | NM_002108.3 |  |  | x |  |  |  |
| HBB | NM_000518.4 |  |  |  | x |  |  |
| HCCS | NM_005333.4 |  |  | x |  |  |  |
| HCFC1 | NM_005334.2 |  |  | x |  |  |  |
| HCN1 | NM_021072.3 |  |  | x |  |  |  |
| HCN2 | NM_001194.3 |  |  | x |  |  |  |
| HDAC4 | NM_006037.3 |  | x | x |  |  |  |
| HDAC8 | NM_018486.2 |  | x | x | x |  |  |
| HEPACAM | NM_152722.4 |  |  | x |  |  |  |
| HES7 | NM_032580.3 |  |  |  | x |  |  |
| HEXA | NM_000520.5 |  |  | x |  |  |  |
| HGD | NM_000187.3 |  |  |  | x |  |  |
| HGF | NM_000601.5 |  |  |  |  | x |  |
| HGSNAT | NM_152419.2 |  |  | x |  |  |  |
| HIVEP2 | NM_006734.3 |  |  | x |  |  |  |
| HK1 | NM_000188.2 | x |  |  |  |  |  |
| HMGA2 | NM_003483.4 |  |  | x |  |  |  |
| HMGCL | NM_000191.2 | x |  |  |  |  |  |
| HNF1A | NM_000545.6 | x |  |  | x |  |  |
| HNF1B | NM_000458.3 | x |  |  | x |  |  |
| HNF4A | NM_175914.4 | x |  |  | x |  |  |
| HNRNPU | NM_031844.2 |  |  | x |  |  |  |
| HOGA1 | NM_138413.3 |  |  |  | x |  |  |
| HOXA1 | NM_005522.4 |  |  | x |  |  |  |
| HOXA13 | NM_000522.4 |  |  |  | x |  |  |
| HOXD13 | NM_000523.3 |  |  |  | x |  |  |
| HPDL | NM_032756.2 |  |  | x |  |  |  |
| HPRT1 | NM_000194.2 |  |  | x | x |  |  |
| HPS1 | NM_000195.4 |  |  |  | x |  |  |
| HPSE2 | NM_021828.4 |  |  |  | x |  |  |
| HRAS | NM_005343.3 |  |  | x | x |  |  |
| HS2ST1 | NM_012262.4 |  |  | x | x |  |  |
| HSD11B2 | NM_000196.3 |  |  |  | x |  |  |
| HSD17B10 | NM_004493.2 |  |  | x |  |  |  |
| HSD17B3 | NM_000197.1 |  |  |  | x |  |  |
| HSD17B4 | NM_000414.3 |  |  |  | x |  |  |
| HSD3B2 | NM_000198.3 |  |  |  | x |  |  |
| HSPA9 | NM_004134.6 |  |  |  | x |  |  |
| HSPG2 | NM_005529.6 |  |  |  | x |  |  |
| HUWE1 | NM_031407.6 |  |  | x |  |  |  |
| HYLS1 | NM_145014.2 |  |  |  | x |  |  |
| ICK | NM_016513.4 |  |  |  | x |  |  |
| IDS | NM_000202.7 |  |  | x |  |  |  |
| IDUA | NM_000203.4 |  |  | x |  |  |  |
| IER3IP1 | NM_016097.4 | x |  |  |  |  |  |
| IFT122 | NM_052985.3 |  |  |  | x |  |  |
| IFT140 | NM_014714.3 |  |  |  | x |  |  |
| IFT172 | NM_015662.2 |  | x | x | x |  |  |
| IFT27 | NM_006860.4 |  | x | x | x |  |  |
| IFT43 | NM_052873.2 |  |  |  | x |  |  |
| IFT52 | NM_001303458.2 |  |  |  | x |  |  |
| IFT57 | NM_018010.3 |  |  |  | x |  |  |
| IFT80 | NM_020800.2 |  |  |  | x |  |  |
| IFT81 | NM_014055.3 |  |  |  | x |  |  |
| IGBP1 | NM_001551.2 |  |  | x |  |  |  |
| IGF1 | NM_000618.4 |  |  | x |  |  |  |
| IGF1R | NM_000875.4 |  |  | x |  |  |  |
| IKBKG | NM_001099857.2 |  |  | x |  |  |  |
| IL1RAPL1 | NM_014271.3 |  |  | x |  |  |  |
| INF2 | NM_022489.3 |  |  |  | x |  |  |
| INPP5E | NM_019892.5 |  | x | x | x |  |  |
| INPPL1 | NM_001567.3 |  |  |  | x |  |  |
| INS | NM_000207.2 | x |  |  |  |  |  |
| INSR | NM_000208.3 | x |  |  | x |  |  |
| INVS | NM_014425.4 |  |  |  | x |  |  |
| IQCB1 | NM_001023570.3 |  |  |  | x |  |  |
| IQSEC1 | NM_014869.6 |  |  | x |  |  |  |
| IQSEC2 | NM_001111125.2 |  | x | x |  |  |  |
| IRF6 | NM_006147.3 |  |  |  | x |  |  |
| ITGA3 | NM_002204.3 |  |  |  | x |  |  |
| ITGA6 | NM_000210.3 |  |  |  | x |  |  |
| ITGA8 | NM_003638.2 |  |  |  | x |  |  |
| ITGB4 | NM_001005731.2 |  |  |  | x |  |  |
| ITPR1 | NM_002222.5 |  |  | x |  |  |  |
| IVD | NM_002225.5 |  |  | x |  |  |  |
| JAG1 | NM_000214.2 |  |  |  | x |  |  |
| JAM3 | NM_032801.4 |  |  |  | x |  |  |
| KANK1 | NM_015158.4 |  |  |  | x |  |  |
| KANK2 | NM_001136191.2 |  |  |  | x |  |  |
| KANK4 | NM_181712.4 |  |  |  | x |  |  |
| KANSL1 | NM_001193466.1 |  |  | x | x |  |  |
| KANSL2 | NM_017822.3 |  |  | x |  |  |  |
| KAT5 | NM_006388.3 |  |  | x |  |  |  |
| KAT6A | NM_006766.4 |  |  | x |  |  |  |
| KAT6B | NM_012330.3 |  |  | x | x |  |  |
| KAT8 | NM_182958.2 |  |  | x |  |  |  |
| KATNAL2 | NM_031303.3 |  |  | x |  |  |  |
| KCNA1 | NM_000217.2 |  |  | x | x |  |  |
| KCNB1 | NM_004975.3 |  |  | x |  |  |  |
| KCNC1 | NM_001112741.1 |  |  | x |  |  |  |
| KCNH1 | NM_172362.2 |  |  | x | x |  |  |
| KCNJ1 | NM_000220.4 |  |  |  | x |  |  |
| KCNJ10 | NM_002241.4 |  |  | x | x |  |  |
| KCNJ11 | NM_000525.3 | x |  |  |  |  |  |
| KCNJ16 | NM_018658.2 |  |  |  | x |  |  |
| KCNJ5 | NM_000890.4 |  |  |  | x |  |  |
| KCNK9 | NM_001282534.1 |  |  | x |  |  |  |
| KCNMA1 | NM_002247.3 |  |  | x |  |  |  |
| KCNN3 | NM_002249.5 |  |  | x |  |  |  |
| KCNQ2 | NM_172107.3 |  |  | x |  |  |  |
| KCNQ3 | NM_004519.3 |  |  | x |  |  |  |
| KCNT1 | NM_020822.2 |  |  | x |  |  |  |
| KCTD1 | NM_001258221.1 |  |  |  | x |  |  |
| KCTD13 | NM_178863.4 |  | x | x |  |  |  |
| KCTD7 | NM_153033.4 |  |  | x |  |  |  |
| KDM1A | NM_001009999.2 |  |  |  | x |  |  |
| KDM3B | NM_016604.3 |  |  | x |  |  |  |
| KDM4B | NM_015015.2 |  |  | x |  |  |  |
| KDM5A | NM_001042603.2 |  |  | x |  |  |  |
| KDM5B | NM_006618.4 |  |  | x |  |  |  |
| KDM5C | NM_004187.3 |  |  | x |  |  |  |
| KDM6A | NM_021140.3 | x | x | x | x |  |  |
| KIAA0556 | NM_015202.3 |  |  |  | x |  |  |
| KIAA0586 | NM_001244189.1 |  |  |  | x |  |  |
| KIAA0753 | NM_014804.2 |  |  |  | x |  |  |
| KIDINS220 | NM_020738.3 |  | x | x |  |  |  |
| KIF11 | NM_004523.3 |  |  | x |  |  |  |
| KIF14 | NM_014875.2 |  |  |  | x |  |  |
| KIF1A | NM_004321.7 |  |  | x |  |  |  |
| KIF7 | NM_198525.2 |  |  |  | x |  |  |
| KIRREL3 | NM_032531.3 |  |  | x |  |  |  |
| KL | NM_004795.3 |  |  |  | x |  |  |
| KLF11 | NM_003597.4 | x |  |  |  |  |  |
| KLF8 | NM_007250.5 |  |  | x |  |  |  |
| KLHL15 | NM_030624.2 |  |  | x |  |  |  |
| KLHL3 | NM_017415.2 |  |  |  | x |  |  |
| KMT2A | NM_001197104.1 |  | x | x |  |  |  |
| KMT2C | NM_170606.2 |  |  | x |  |  |  |
| KMT2D | NM_003482.3 | x | x | x | x |  |  |
| KMT2E | NM_182931.2 |  |  | x |  |  |  |
| KMT5B | NM_017635.4 |  |  | x |  |  |  |
| KRAS | NM_004985.4 |  |  | x | x |  |  |
| KSR2 | NM_173598.4 |  | x |  |  |  |  |
| KYNU | NM_003937.2 |  |  |  | x |  |  |
| L1CAM | NM_000425.4 |  |  | x |  |  |  |
| LAMB2 | NM_002292.3 |  |  |  | x |  |  |
| LAMB3 | NM_000228.2 |  |  |  | x |  |  |
| LAMC2 | NM_005562.2 |  |  |  | x |  |  |
| LAMC3 | NM_006059.3 |  |  | x |  |  |  |
| LAMP2 | NM_002294.2 |  |  | x |  |  |  |
| LARS1 | NM_020117.10 |  |  |  | x |  |  |
| LAS1L | NM_031206.4 |  | x | x |  |  |  |
| LCAT | NM_000229.1 |  |  |  | x |  |  |
| LDHA | NM_005566.3 |  |  |  | x |  |  |
| LEP | NM_000230.2 |  | x |  |  |  |  |
| LEPR | NM_002303.5 |  | x |  |  |  |  |
| LFNG | NM_001040167.1 |  |  |  | x |  |  |
| LGI1 | NM_005097.3 |  |  | x |  |  |  |
| LIMK1 | NM_002314.3 |  |  | x |  |  |  |
| LMBRD1 | NM_018368.3 |  |  |  | x |  |  |
| LMNA | NM_170707.3 |  |  |  | x |  |  |
| LMNB1 | NM_005573.3 |  |  | x |  |  |  |
| LMX1B | NM_002316.3 |  |  |  | x |  |  |
| LONP1 | NM_004793.3 |  |  |  | x |  |  |
| LPIN1 | NM_145693.3 |  |  |  | x |  |  |
| LRBA | NM_006726.4 | x |  |  |  |  |  |
| LRIG2 | NM_014813.2 |  |  |  | x |  |  |
| LRP2 | NM_004525.2 |  |  | x | x |  |  |
| LRP4 | NM_002334.3 |  |  |  | x |  |  |
| LRP6 | NM_002336.2 | x |  |  |  |  |  |
| LRRC43 | NM_001098519.1 |  |  | x |  |  |  |
| LTBP4 | NM_003573.2 |  |  |  | x |  |  |
| LYZ | NM_000239.2 |  |  |  | x |  |  |
| LZTFL1 | NM_020347.3 |  | x | x | x |  |  |
| LZTR1 | NM_006767.3 |  |  | x |  |  |  |
| MAFA | NM_201589.3 | x |  |  |  |  |  |
| MAFB | NM_005461.4 |  |  |  | x |  |  |
| MAGED2 | NM_177433.2 |  |  |  | x |  |  |
| MAGEL2 | NM_019066.4 |  | x | x |  |  |  |
| MAGI2 | NM_012301.3 |  |  | x |  |  |  |
| MAN1B1 | NM_016219.4 |  |  | x |  |  |  |
| MAN2B1 | NM_000528.3 |  |  | x |  |  |  |
| MANBA | NM_005908.3 |  |  | x |  |  |  |
| MAOA | NM_000240.3 |  |  | x |  |  |  |
| MAP2K1 | NM_002755.3 |  |  | x | x |  |  |
| MAP2K2 | NM_030662.3 |  |  | x | x |  |  |
| MAPK1 | NM_002745.4 |  |  | x |  |  |  |
| MAPK8IP3 | NM_001040439.1 |  |  | x |  |  |  |
| MAPRE2 | NM_014268.3 |  |  |  | x |  |  |
| MAST1 | NM_014975.2 |  |  | x |  |  |  |
| MBD5 | NM_018328.4 |  |  | x |  |  |  |
| MBOAT7 | NM_024298.4 |  |  | x |  |  |  |
| MBTPS2 | NM_015884.3 |  |  | x | x |  |  |
| MC3R | NM_019888.3 |  | x |  |  |  |  |
| MC4R | NM_005912.2 |  | x |  |  |  |  |
| MCPH1 | NM_024596.4 |  |  | x |  |  |  |
| MECP2 | NM_004992.3 |  | x | x |  |  |  |
| MED12 | NM_005120.2 |  |  | x |  |  |  |
| MED13L | NM_015335.4 |  |  | x |  |  |  |
| MED17 | NM_004268.4 |  |  | x |  |  |  |
| MED23 | NM_015979.3 |  |  | x |  |  |  |
| MEF2C | NM_002397.4 |  |  | x |  |  |  |
| MEFV | NM_000243.2 |  |  |  | x |  |  |
| MEGF8 | NM_001410.2 |  | x | x |  |  |  |
| MEIS2 | NM_170674.4 |  |  | x |  |  |  |
| MESP2 | NM_001039958.1 |  |  |  | x |  |  |
| MET | NM_001127500.2 |  |  | x |  | x |  |
| METTL5 | NM_014168.3 |  |  | x |  |  |  |
| MFSD8 | NM_152778.2 |  |  | x |  |  |  |
| MGP | NM_000900.4 |  |  |  | x |  |  |
| MIB1 | NM_020774.3 |  |  | x |  |  |  |
| MID1 | NM_000381.3 |  |  | x |  |  |  |
| MIR17HG | NR_027350.1 |  |  |  | x |  |  |
| MKKS | NM_018848.3 |  | x | x | x |  |  |
| MKS1 | NM_017777.3 |  | x | x | x |  |  |
| MLH1 | NM_000249.3 |  |  |  | x |  |  |
| MMACHC | NM_015506.2 |  |  | x | x |  |  |
| MMUT | NM_000255.3 |  |  | x | x |  |  |
| MN1 | NM_002430.2 |  |  | x |  |  |  |
| MNX1 | NM_005515.3 | x |  |  | x |  |  |
| MPV17 | NM_002437.4 | x |  |  |  |  |  |
| MRAP2 | NM_138409.3 | x | x |  |  |  |  |
| MRAS | NM_012219.4 |  |  | x |  |  |  |
| MRPS22 | NM_020191.2 |  |  |  | x |  |  |
| MSH2 | NM_000251.2 |  |  |  | x |  |  |
| MSH6 | NM_000179.2 |  |  |  | x |  |  |
| MSL3 | NM_001282174.1 |  |  | x |  |  |  |
| MTHFR | NM_005957.4 |  |  | x |  |  |  |
| MTM1 | NM_000252.2 |  |  |  | x |  |  |
| MTR | NM_000254.2 |  |  | x |  |  |  |
| MUC1 | NM_002456.5 |  |  |  | x |  |  |
| MVK | NM_000431.3 |  |  |  | x |  |  |
| MYCN | NM_005378.5 |  |  | x | x |  |  |
| MYH9 | NM_002473.5 |  |  |  | x |  |  |
| MYO1E | NM_004998.3 |  |  |  | x |  |  |
| MYT1L | NM_015025.3 |  | x | x |  |  |  |
| NAA10 | NM_003491.3 |  |  | x | x |  |  |
| NAA15 | NM_057175.4 |  |  | x |  |  |  |
| NAGLU | NM_000263.3 |  |  | x |  |  |  |
| NARS1 | NM_004539.3 |  |  | x |  |  |  |
| NARS2 | NM_024678.5 |  |  |  | x |  |  |
| NBN | NM_002485.4 |  |  |  | x |  |  |
| NCOA1 | NM_003743.4 |  | x |  |  |  |  |
| NDE1 | NM_001143979.1 |  |  | x |  |  |  |
| NDST1 | NM_001543.4 |  |  | x |  |  |  |
| NDUFA1 | NM_004541.3 |  |  | x |  |  |  |
| NDUFAF8 | NM_001086521.1 |  |  | x |  |  |  |
| NECAP1 | NM_015509.3 |  |  | x |  |  |  |
| NECTIN1 | NM_002855.4 |  |  |  | x |  |  |
| NEDD4L | NM_015277.5 |  |  | x |  |  |  |
| NEK1 | NM_012224.2 |  |  |  | x |  |  |
| NEK8 | NM_178170.2 |  |  |  | x |  |  |
| NEUROD1 | NM_002500.4 | x |  |  |  |  |  |
| NEUROG3 | NM_020999.3 | x |  |  |  |  |  |
| NEXMIF | NM_001008537.2 |  |  | x | x |  |  |
| NF1 | NM_000267.3 |  |  | x | x |  |  |
| NFIB | NM_001190737.1 |  |  | x |  |  |  |
| NFIX | NM_001271043.2 |  |  | x |  |  |  |
| NGFR | NM_002507.3 |  |  | x |  |  |  |
| NHLRC1 | NM_198586.2 |  |  | x |  |  |  |
| NHP2 | NM_017838.3 |  |  |  | x |  |  |
| NHS | NM_198270.3 |  |  | x |  |  |  |
| NIPBL | NM_133433.3 |  | x | x | x |  |  |
| NKAP | NM_024528.3 |  |  | x |  |  |  |
| NKX2-2 | NM_002509.3 | x |  |  |  |  |  |
| NLGN3 | NM_018977.3 |  |  | x |  |  |  |
| NLGN4X | NM_020742.3 |  |  | x |  |  |  |
| NLRP3 | NM_004895.4 |  |  |  | x |  |  |
| NOTCH2 | NM_024408.3 |  |  |  | x |  |  |
| NOTCH3 | NM_000435.2 |  |  |  | x |  |  |
| NPC1 | NM_000271.4 |  |  | x |  |  |  |
| NPC2 | NM_006432.3 |  |  | x |  |  |  |
| NPHP1 | NM_000272.3 |  | x | x | x |  |  |
| NPHP3 | NM_153240.4 |  |  |  | x |  |  |
| NPHP4 | NM_015102.4 |  |  |  | x |  |  |
| NPHS1 | NM_004646.3 |  |  |  | x |  |  |
| NPHS2 | NM_014625.3 |  |  |  | x |  |  |
| NR0B1 | NM_000475.4 |  |  |  | x |  |  |
| NR3C2 | NM_000901.4 |  |  |  | x |  |  |
| NRAS | NM_002524.4 |  |  | x |  |  |  |
| NRIP1 | NM_003489.3 |  |  |  | x |  |  |
| NRP1 | NM_003873.5 |  | x |  |  |  |  |
| NRP2 | NM_201266.1 |  | x |  |  |  |  |
| NRXN1 | NM_001135659.2 |  |  | x |  |  |  |
| NRXN2 | NM_138732.2 |  |  | x |  |  |  |
| NSD1 | NM_022455.4 |  |  | x | x |  |  |
| NSDHL | NM_015922.2 |  |  | x | x |  |  |
| NSUN2 | NM_017755.5 |  |  | x |  |  |  |
| NTNG1 | NM_014917.3 |  |  | x |  |  |  |
| NTNG2 | NM_032536.3 |  |  | x |  |  |  |
| NTRK2 | NM_006180.4 |  | x |  |  |  |  |
| NUP107 | NM_020401.3 |  |  |  | x |  |  |
| NUP205 | NM_015135.2 |  |  |  | x |  |  |
| NUP93 | NM_014669.4 |  |  |  | x |  |  |
| OCLN | NM_002538.3 |  |  |  | x |  |  |
| OCRL | NM_000276.3 |  |  | x | x |  |  |
| OFD1 | NM_003611.2 |  |  | x | x |  |  |
| OPHN1 | NM_002547.2 |  |  | x |  |  |  |
| OPLAH | NM_017570.4 |  |  |  | x |  |  |
| OSR1 | NM_145260.2 |  |  |  | x |  |  |
| OTC | NM_000531.5 |  |  | x |  |  |  |
| OXR1 | NM_001198534.1 |  |  | x |  |  |  |
| OXTR | NM_000916.3 |  |  | x |  |  |  |
| PACS1 | NM_018026.3 |  |  | x |  |  |  |
| PACS2 | NM_001100913.2 |  |  | x |  |  |  |
| PAFAH1B1 | NM_000430.3 |  |  | x |  |  |  |
| PAH | NM_000277.2 |  |  | x |  |  |  |
| PAK3 | NM_002578.4 |  |  | x |  |  |  |
| PALB2 | NM_024675.3 |  |  |  | x |  |  |
| PAX2 | NM_003987.4 |  |  |  | x |  |  |
| PAX4 | NM_001366110.1 | x |  |  |  |  |  |
| PAX5 | NM_016734.2 |  |  | x |  |  |  |
| PAX6 | NM_000280.4 | x |  |  |  |  |  |
| PBX1 | NM_002585.3 |  |  |  | x |  |  |
| PC | NM_000920.3 |  |  |  | x |  |  |
| PCBD1 | NM_000281.3 | x |  |  |  |  |  |
| PCCA | NM_000282.3 |  |  | x |  |  |  |
| PCCB | NM_000532.4 |  |  | x |  |  |  |
| PCDH19 | NM_001184880.1 |  |  | x |  |  |  |
| PCSK1 | NM_000439.4 |  | x |  |  |  |  |
| PDE1A | NM_005019.4 |  |  |  | x |  |  |
| PDE6D | NM_002601.3 |  |  |  | x |  |  |
| PDHA1 | NM_000284.3 |  |  | x |  |  |  |
| PDHX | NM_003477.2 |  |  | x |  |  |  |
| PDSS1 | NM_014317.4 |  |  |  | x |  |  |
| PDSS2 | NM_020381.3 |  |  |  | x |  |  |
| PDX1 | NM_000209.3 | x |  |  |  |  |  |
| PET100 | NM_001171155.1 |  |  |  | x |  |  |
| PEX1 | NM_000466.2 |  |  | x | x |  |  |
| PEX10 | NM_153818.1 |  |  |  | x |  |  |
| PEX11B | NM_003846.2 |  |  |  | x |  |  |
| PEX12 | NM_000286.2 |  |  | x | x |  |  |
| PEX13 | NM_002618.3 |  |  |  | x |  |  |
| PEX14 | NM_004565.2 |  |  |  | x |  |  |
| PEX16 | NM_004813.2 |  |  |  | x |  |  |
| PEX19 | NM_002857.3 |  |  |  | x |  |  |
| PEX2 | NM_000318.2 |  |  |  | x |  |  |
| PEX26 | NM_017929.5 |  |  |  | x |  |  |
| PEX3 | NM_003630.2 |  |  |  | x |  |  |
| PEX5 | NM_001131025.1 |  |  |  | x |  |  |
| PEX6 | NM_000287.3 |  |  | x | x |  |  |
| PEX7 | NM_000288.3 |  |  | x |  |  |  |
| PGAP2 | NM_001256240.1 |  |  | x |  |  |  |
| PGAP3 | NM_033419.4 |  |  | x |  |  |  |
| PGK1 | NM_000291.3 |  |  |  | x |  |  |
| PGM1 | NM_002633.2 | x |  |  |  |  |  |
| PGM3 | NM_001199917.1 |  |  |  | x |  |  |
| PHEX | NM_000444.5 |  |  |  | x |  |  |
| PHF21A | NM_001101802.1 |  |  | x |  |  |  |
| PHF6 | NM_032458.2 |  | x | x | x |  |  |
| PHF8 | NM_015107.2 |  |  | x |  |  |  |
| PHGDH | NM_006623.3 |  |  | x | x |  |  |
| PHIP | NM_017934.6 |  | x | x |  |  |  |
| PIEZO2 | NM_022068.3 |  |  |  | x |  |  |
| PIGA | NM_002641.3 |  |  | x | x |  |  |
| PIGK | NM_005482.2 |  |  | x |  |  |  |
| PIGL | NM_004278.3 |  |  |  | x |  |  |
| PIGN | NM_176787.4 |  |  | x | x |  |  |
| PIGO | NM_032634.3 |  |  | x |  |  |  |
| PIGT | NM_015937.5 |  |  |  | x |  |  |
| PIGV | NM_017837.3 |  |  | x |  |  |  |
| PIK3CA | NM_006218.3 |  |  |  | x |  |  |
| PIK3R2 | NM_005027.3 |  |  |  | x |  |  |
| PITX2 | NM_153427.2 |  |  |  | x |  |  |
| PKD1 | NM_001009944.2 |  |  |  | x |  |  |
| PKD2 | NM_000297.3 |  |  |  | x |  |  |
| PKHD1 | NM_138694.3 |  |  |  | x |  |  |
| PLCB1 | NM_015192.3 |  |  | x |  |  |  |
| PLCE1 | NM_016341.3 |  |  |  | x |  |  |
| PLG | NM_000301.3 |  |  |  | x |  |  |
| PLOD1 | NM_000302.3 |  |  |  | x |  |  |
| PLP1 | NM_000533.4 |  |  | x |  |  |  |
| PLXNA1 | NM_032242.3 |  | x |  |  |  |  |
| PLXNA2 | NM_025179.3 |  | x |  |  |  |  |
| PLXNA3 | NM_017514.4 |  | x |  |  |  |  |
| PLXNA4 | NM_020911.1 |  | x |  |  |  |  |
| PMM2 | NM_000303.2 | x |  | x | x |  |  |
| PMPCB | NM_004279.2 |  |  | x |  |  |  |
| PNKP | NM_007254.3 |  |  | x |  |  |  |
| PNPLA6 | NM_006702.4 |  |  |  | x |  |  |
| PNPO | NM_018129.3 |  |  | x |  |  |  |
| POC1A | NM_015426.4 |  |  |  | x |  |  |
| POC1B | NM_172240.2 |  |  |  | x |  |  |
| POGZ | NM_015100.3 |  |  | x |  |  |  |
| POLG | NM_002693.2 |  |  | x |  |  |  |
| POLR2A | NM_000937.4 |  |  | x |  |  |  |
| POMC | NM_001035256.2 |  | x |  | x |  |  |
| POMGNT1 | NM_017739.3 |  |  | x |  |  |  |
| POMT1 | NM_007171.3 |  |  |  | x |  |  |
| POR | NM_000941.2 |  |  |  | x |  |  |
| PORCN | NM_203475.2 |  |  | x | x |  |  |
| POU1F1 | NM_000306.3 |  |  | x |  |  |  |
| POU3F3 | NM_006236.2 |  |  | x |  |  |  |
| PPP1CB | NM_206876.1 |  |  | x |  |  |  |
| PPP1R15B | NM_032833.4 | x |  |  | x |  |  |
| PPP2CA | NM_002715.3 |  |  | x |  |  |  |
| PPP2R5D | NM_006245.3 |  |  | x |  |  |  |
| PPT1 | NM_000310.3 |  |  | x |  |  |  |
| PQBP1 | NM_005710.2 |  |  | x | x |  |  |
| PRICKLE1 | NM_153026.2 |  |  | x |  |  |  |
| PRKCD | NM_006254.3 |  |  |  | x |  |  |
| PRKRA | NM_003690.4 |  |  | x |  |  |  |
| PRMT7 | NM_019023.3 |  | x | x |  |  |  |
| PRODH | NM_016335.4 |  |  | x | x |  |  |
| PROK2 | NM_001126128.1 |  | x | x |  |  |  |
| PROKR2 | NM_144773.3 |  | x | x | x |  |  |
| PRPS1 | NM_002764.3 |  |  | x | x |  |  |
| PRRT2 | NM_145239.2 |  |  | x |  |  |  |
| PRSS12 | NM_003619.3 |  |  | x |  |  |  |
| PSAP | NM_002778.3 |  |  | x | x |  |  |
| PTCHD1 | NM_173495.2 |  |  | x |  |  |  |
| PTEN | NM_000314.6 |  |  | x | x |  |  |
| PTF1A | NM_178161.2 | x |  |  |  |  |  |
| PTH | NM_000315.3 |  |  |  | x |  |  |
| PTH1R | NM_000316.2 |  |  |  | x |  |  |
| PTPN11 | NM_002834.4 |  |  | x | x |  |  |
| PTPRO | NM_030667.2 |  |  |  | x |  |  |
| PTS | NM_000317.2 |  |  | x |  |  |  |
| PUF60 | NM_078480.2 |  |  | x | x |  |  |
| PURA | NM_005859.4 |  |  | x |  |  |  |
| PYGM | NM_005609.3 |  |  |  | x |  |  |
| QARS1 | NM_005051.2 |  |  | x |  |  |  |
| QRICH1 | NM_017730.3 |  |  | x |  |  |  |
| RAB18 | NM_021252.4 |  |  |  | x |  |  |
| RAB23 | NM_183227.2 |  | x | x | x |  |  |
| RAB39B | NM_171998.3 |  |  | x |  |  |  |
| RAB3GAP1 | NM_012233.2 |  |  |  | x |  |  |
| RAB3GAP2 | NM_012414.3 |  |  |  | x |  |  |
| RAD21 | NM_006265.2 |  | x | x |  |  |  |
| RAD51C | NM_058216.2 |  |  |  | x |  |  |
| RAF1 | NM_002880.3 |  |  | x |  |  |  |
| RAI1 | NM_030665.3 |  | x | x | x |  |  |
| RALGAPA1 | NM_014990.2 |  |  | x |  |  |  |
| RAP1A | NM_001010935.2 |  |  |  | x |  |  |
| RAP1B | NM_015646.5 |  |  |  | x |  |  |
| RASA1 | NM_002890.2 |  |  | x |  |  |  |
| RBBP8 | NM_002894.2 |  |  |  | x |  |  |
| RBM10 | NM_005676.4 |  |  | x | x |  |  |
| RBM8A | NM_005105.4 |  |  |  | x |  |  |
| RECQL4 | NM_004260.3 |  |  |  | x |  |  |
| RELN | NM_005045.3 |  |  | x |  |  |  |
| REN | NM_000537.3 |  |  |  | x |  |  |
| RERE | NM_012102.3 |  |  |  | x |  |  |
| RET | NM_020975.5 |  |  |  | x |  |  |
| RFX6 | NM_173560.3 | x |  |  |  |  |  |
| RIMS2 | NM_014677.4 | x |  | x |  |  |  |
| RIN2 | NM_018993.3 |  |  |  | x |  |  |
| RIPK4 | NM_020639.2 |  |  |  | x |  |  |
| RIPPLY2 | NM_001009994.2 |  |  |  | x |  |  |
| RIT1 | NM_006912.5 |  |  | x |  |  |  |
| RLIM | NM_183353.2 |  |  | x |  |  |  |
| RMND1 | NM_017909.3 |  |  |  | x |  |  |
| RNF135 | NM_032322.3 |  |  | x |  |  |  |
| RNU4ATAC | NR_023343.1 |  |  |  | x |  |  |
| ROBO2 | NM_002942.4 |  |  |  | x |  |  |
| ROR2 | NM_004560.3 |  |  |  | x |  |  |
| RORA | NM_134261.2 |  |  | x |  |  |  |
| RPGRIP1L | NM_015272.4 |  |  |  | x |  |  |
| RPL10 | NM_006013.4 |  |  | x |  |  |  |
| RPL11 | NM_000975.4 |  |  |  | x |  |  |
| RPL26 | NM_000987.4 |  |  |  | x |  |  |
| RPL35A | NM_000996.3 |  |  |  | x |  |  |
| RPL5 | NM_000969.4 |  |  |  | x |  |  |
| RPS10 | NM_001014.4 |  |  |  | x |  |  |
| RPS17 | NM_001021.6 |  |  |  | x |  |  |
| RPS19 | NM_001022.3 |  |  |  | x |  |  |
| RPS24 | NM_033022.3 |  |  |  | x |  |  |
| RPS26 | NM_001029.4 |  |  |  | x |  |  |
| RPS28 | NM_001031.4 |  |  |  | x |  |  |
| RPS29 | NM_001032.4 |  |  |  | x |  |  |
| RPS6KA3 | NM_004586.2 |  | x | x |  |  |  |
| RPS7 | NM_001011.3 |  |  |  | x |  |  |
| RRAS | NM_006270.4 |  |  | x |  |  |  |
| RRAS2 | NM_012250.5 |  |  | x |  |  |  |
| RRM2B | NM_015713.4 |  |  |  | x |  |  |
| RTTN | NM_173630.3 |  |  |  | x |  |  |
| SALL1 | NM_002968.2 |  |  |  | x |  |  |
| SALL4 | NM_020436.4 |  |  |  | x |  |  |
| SARS2 | NM_017827.3 |  |  |  | x |  |  |
| SATB2 | NM_015265.3 |  |  | x |  |  |  |
| SBDS | NM_016038.3 |  |  |  | x |  |  |
| SC5D | NM_006918.4 |  |  |  | x |  |  |
| SCAF4 | NM_020706.2 |  |  | x |  |  |  |
| SCARB2 | NM_005506.3 |  |  | x | x |  |  |
| SCLT1 | NM_144643.3 |  |  |  | x |  |  |
| SCN10A | NM_006514.3 |  |  |  | x |  |  |
| SCN1A | NM_001165963.2 |  |  | x |  |  |  |
| SCN1B | NM_001037.4 |  |  | x |  |  |  |
| SCN2A | NM_021007.3 |  |  | x |  |  |  |
| SCN4A | NM_000334.4 |  |  |  | x |  |  |
| SCN8A | NM_014191.3 |  |  | x |  |  |  |
| SCNN1A | NM_001038.5 |  |  |  | x |  |  |
| SCNN1B | NM_000336.2 |  |  |  | x |  |  |
| SCNN1G | NM_001039.3 |  |  |  | x |  |  |
| SCO1 | NM_004589.3 |  |  |  | x |  |  |
| SDCCAG8 | NM_006642.4 |  | x | x | x |  |  |
| SDHB | NM_003000.2 |  |  |  | x |  |  |
| SDHC | NM_003001.3 |  |  |  | x |  |  |
| SDHD | NM_003002.3 |  |  |  | x |  |  |
| SEMA3A | NM_006080.2 |  | x |  |  |  |  |
| SEMA3B | NM_004636.3 |  | x |  |  |  |  |
| SEMA3C | NM_006379.4 |  | x |  |  |  |  |
| SEMA3D | NM_152754.2 |  | x |  |  |  |  |
| SEMA3E | NM_012431.2 |  | x |  | x |  |  |
| SEMA3F | NM_004186.4 |  | x |  |  |  |  |
| SEMA3G | NM_020163.2 |  | x |  |  |  |  |
| SEMA5A | NM_003966.2 |  |  | x |  |  |  |
| SERPINH1 | NM_001235.3 |  |  |  | x |  |  |
| SET | NM_001122821.1 |  |  | x |  |  |  |
| SETBP1 | NM_015559.2 |  |  | x | x |  |  |
| SETD1A | NM_014712.2 |  |  | x |  |  |  |
| SETD2 | NM_014159.6 |  |  | x |  |  |  |
| SETD5 | NM_001080517.2 |  | x | x |  |  |  |
| SF3B4 | NM_005850.4 |  |  |  | x |  |  |
| SGK1 | NM_005627.3 |  |  |  | x |  |  |
| SGSH | NM_000199.4 |  |  | x |  |  |  |
| SH2B1 | NM_001145795.1 |  | x |  |  |  |  |
| SHANK1 | NM_016148.4 |  |  | x |  |  |  |
| SHANK2 | NM_012309.4 |  |  | x |  |  |  |
| SHANK3 | NM_001372044.1 |  |  | x |  |  |  |
| SHH | NM_000193.3 |  |  |  | x |  |  |
| SHOC2 | NM_007373.3 |  |  | x |  |  |  |
| SI | NM_001041.3 |  |  |  | x |  |  |
| SIM1 | NM_005068.2 |  | x |  |  |  |  |
| SIX1 | NM_005982.3 |  |  |  | x |  |  |
| SIX2 | NM_016932.4 |  |  |  | x |  |  |
| SIX5 | NM_175875.4 |  |  |  | x |  |  |
| SKI | NM_003036.3 |  |  | x |  |  |  |
| SLC12A1 | NM_000338.2 |  |  |  | x |  |  |
| SLC12A3 | NM_000339.2 |  |  |  | x |  |  |
| SLC13A5 | NM_177550.4 |  |  | x |  |  |  |
| SLC16A1 | NM_003051.3 | x |  |  |  |  |  |
| SLC16A12 | NM_213606.3 |  |  |  | x |  |  |
| SLC16A2 | NM_006517.4 |  |  | x |  |  |  |
| SLC17A5 | NM_012434.4 |  |  | x |  |  |  |
| SLC19A2 | NM_006996.2 | x |  |  |  |  |  |
| SLC1A1 | NM_004170.5 |  |  | x | x |  |  |
| SLC22A12 | NM_144585.3 |  |  |  | x |  |  |
| SLC22A5 | NM_003060.3 | x |  |  |  |  |  |
| SLC25A1 | NM_005984.4 |  |  |  | x |  |  |
| SLC25A13 | NM_014251.2 |  |  | x |  |  |  |
| SLC25A15 | NM_014252.3 |  |  | x |  |  |  |
| SLC25A20 | NM_000387.5 | x |  |  |  |  |  |
| SLC25A22 | NM_024698.5 |  |  | x |  |  |  |
| SLC26A1 | NM_213613.3 |  |  |  | x |  |  |
| SLC26A4 | NM_000441.1 |  |  |  | x |  |  |
| SLC26A6 | NM_022911.2 |  |  |  | x |  |  |
| SLC2A1 | NM_006516.2 |  |  | x |  |  |  |
| SLC2A10 | NM_030777.3 |  |  |  | x |  |  |
| SLC2A2 | NM_000340.1 | x |  |  | x |  |  |
| SLC2A9 | NM_020041.2 |  |  |  | x |  |  |
| SLC34A1 | NM_003052.4 |  |  |  | x |  |  |
| SLC34A3 | NM_080877.2 |  |  |  | x |  |  |
| SLC35A2 | NM_001042498.2 |  |  | x |  |  |  |
| SLC35A3 | NM_012243.2 |  |  | x |  |  |  |
| SLC35C1 | NM_018389.4 |  |  | x |  |  |  |
| SLC36A2 | NM_181776.2 |  |  |  | x |  |  |
| SLC37A4 | NM_001164277.1 |  |  |  | x |  |  |
| SLC3A1 | NM_000341.3 |  |  |  | x |  |  |
| SLC41A1 | NM_173854.5 |  |  |  | x |  |  |
| SLC45A1 | NM_001080397.2 |  |  | x |  |  |  |
| SLC46A1 | NM_080669.5 |  |  | x |  |  |  |
| SLC4A1 | NM_000342.3 |  |  |  | x |  |  |
| SLC4A4 | NM_003759.3 |  |  |  | x |  |  |
| SLC5A2 | NM_003041.3 |  |  |  | x |  |  |
| SLC6A1 | NM_003042.3 |  |  | x |  |  |  |
| SLC6A19 | NM_001003841.2 |  |  |  | x |  |  |
| SLC6A20 | NM_020208.3 |  |  |  | x |  |  |
| SLC6A4 | NM_001045.5 |  |  | x |  |  |  |
| SLC6A8 | NM_005629.3 |  |  | x |  |  |  |
| SLC7A7 | NM_001126106.2 |  |  |  | x |  |  |
| SLC7A9 | NM_014270.4 |  |  |  | x |  |  |
| SLC9A3R1 | NM_004252.4 |  |  |  | x |  |  |
| SLC9A6 | NM_006359.2 |  |  | x |  |  |  |
| SLIT2 | NM_004787.3 |  |  |  | x |  |  |
| SLX4 | NM_032444.3 |  |  |  | x |  |  |
| SMAD3 | NM_005902.3 |  |  |  | x |  |  |
| SMAD4 | NM_005359.5 |  |  | x |  |  |  |
| SMARCA2 | NM_003070.4 |  |  | x |  |  |  |
| SMARCA4 | NM_001128849.1 |  |  | x |  |  |  |
| SMARCAL1 | NM_014140.3 |  |  |  | x |  |  |
| SMARCB1 | NM_003073.4 |  |  | x |  |  |  |
| SMARCC2 | NM_003075.4 |  |  | x |  |  |  |
| SMARCE1 | NM_003079.4 |  |  | x | x |  |  |
| SMC1A | NM_006306.3 |  | x | x | x |  |  |
| SMC3 | NM_005445.3 |  | x | x |  |  |  |
| SMG8 | NM_018149.6 |  |  | x |  |  |  |
| SMO | NM_005631.4 |  |  | x |  |  |  |
| SMOC1 | NM_001034852.2 |  |  |  | x |  |  |
| SMPD4 | NM_017951.4 |  |  | x |  |  |  |
| SMS | NM_004595.4 |  |  | x |  |  |  |
| SOBP | NM_018013.3 |  |  | x |  |  |  |
| SON | NM_032195.2 |  |  | x |  |  |  |
| SOS1 | NM_005633.3 |  |  | x |  |  |  |
| SOS2 | NM_006939.3 |  |  | x |  |  |  |
| SOX10 | NM_006941.3 |  | x | x |  |  |  |
| SOX11 | NM_003108.3 |  |  | x | x |  |  |
| SOX17 | NM_022454.3 |  |  |  | x |  |  |
| SOX18 | NM_018419.2 |  |  |  | x | x |  |
| SOX3 | NM_005634.2 |  |  | x |  |  |  |
| SOX5 | NM_006940.5 |  |  | x |  |  |  |
| SOX6 | NM_033326.3 |  |  | x |  |  |  |
| SOX9 | NM_000346.3 |  |  |  | x |  |  |
| SPECC1L | NM_015330.4 |  |  |  | x |  |  |
| SPINT2 | NM_021102.3 |  |  |  | x |  |  |
| SPP1 | NM_001040058.1 |  |  |  | x |  |  |
| SPRED1 | NM_152594.2 |  |  | x |  |  |  |
| SPTAN1 | NM_001130438.2 |  |  | x |  |  |  |
| SRCAP | NM_006662.2 |  |  | x | x |  |  |
| SRD5A3 | NM_024592.4 |  |  | x |  |  |  |
| SRGAP1 | NM_020762.3 |  |  |  | x |  |  |
| SRGAP3 | NM_014850.3 |  |  | x |  |  |  |
| ST3GAL3 | NM_006279.4 |  |  | x |  |  |  |
| ST3GAL5 | NM_003896.3 |  |  | x |  |  |  |
| STAG1 | NM_005862.2 |  |  | x |  |  |  |
| STAMBP | NM_006463.5 |  |  | x |  |  |  |
| STAR | NM_000349.2 |  |  |  | x |  |  |
| STAT3 | NM_139276.2 | x |  |  |  |  |  |
| STIL | NM_003035.2 |  |  | x |  |  |  |
| STK11 | NM_000455.4 |  |  |  | x |  |  |
| STK39 | NM_013233.2 |  |  |  | x |  |  |
| STRA6 | NM_022369.3 |  |  |  | x |  |  |
| STRADA | NM_001003787.2 |  |  |  | x |  |  |
| STUB1 | NM_005861.3 |  |  |  | x |  |  |
| STX16 | NM_001001433.2 |  | x | x | x |  |  |
| STX1A | NM_004603.3 |  |  | x |  |  |  |
| STX1B | NM_052874.4 |  |  | x |  |  |  |
| STXBP1 | NM_003165.3 |  |  | x |  |  |  |
| SUCLA2 | NM_003850.2 |  |  |  | x |  |  |
| SUGCT | NM_024728.2 |  |  |  | x |  |  |
| SYN1 | NM_133499.2 |  |  | x |  |  |  |
| SYNE1 | NM_033071.3 |  |  | x |  |  |  |
| SYNGAP1 | NM_006772.2 |  |  | x |  |  |  |
| SYNJ1 | NM_003895.3 |  |  | x |  |  |  |
| SYNPO2 | NM_133477.2 |  |  |  | x |  |  |
| SYP | NM_003179.2 |  |  | x |  |  |  |
| SZT2 | NM_015284.3 |  |  | x |  |  |  |
| TAB2 | NM_015093.5 |  |  | x |  |  |  |
| TACO1 | NM_016360.3 |  |  |  | x |  |  |
| TAF1 | NM_004606.4 |  |  | x |  |  |  |
| TAOK1 | NM_020791.2 |  |  | x |  |  |  |
| TAOK2 | NM_016151.3 |  |  | x |  |  |  |
| TAPT1 | NM_153365.2 |  |  |  | x |  |  |
| TBC1D20 | NM_144628.3 |  |  |  | x |  |  |
| TBC1D24 | NM_001199107.1 |  |  | x | x |  |  |
| TBC1D8B | NM_017752.2 |  |  |  | x |  |  |
| TBCE | NM_003193.4 |  |  |  | x |  |  |
| TBL1XR1 | NM_024665.5 |  |  | x |  |  |  |
| TBR1 | NM_006593.3 |  |  | x |  |  |  |
| TBX1 | NM_080647.1 |  | x | x |  |  |  |
| TBX18 | NM_001080508.2 |  |  |  | x |  |  |
| TBX3 | NM_005996.3 |  | x | x |  |  |  |
| TCF20 | NM_005650.3 |  |  | x |  |  |  |
| TCF4 | NM_001083962.1 |  |  | x |  |  |  |
| TCTN1 | NM_001082538.2 |  |  |  | x |  |  |
| TCTN2 | NM_024809.4 |  |  |  | x |  |  |
| TCTN3 | NM_015631.5 |  |  |  | x |  |  |
| TECR | NM_138501.5 |  |  | x |  |  |  |
| TERC | NR_001566.1 |  |  |  | x |  |  |
| TET2 | NM_001127208.2 |  |  | x |  |  |  |
| TFAP2A | NM_001372066.1 |  |  |  | x |  |  |
| THOC6 | NM_024339.4 |  |  | x | x |  |  |
| THRA | NM_199334.3 |  | x | x |  |  |  |
| TIMM8A | NM_004085.3 |  |  | x |  |  |  |
| TLK2 | NM_006852.3 |  |  | x |  |  |  |
| TMCO1 | NM_019026.4 |  |  |  | x |  |  |
| TMEM107 | NM_032354.4 |  |  |  | x |  |  |
| TMEM138 | NM_016464.4 |  |  |  | x |  |  |
| TMEM216 | NM_001173990.2 |  |  |  | x |  |  |
| TMEM231 | NM_001077416.2 |  |  |  | x |  |  |
| TMEM237 | NM_001044385.2 |  |  |  | x |  |  |
| TMEM67 | NM_153704.5 |  |  |  | x |  |  |
| TMEM70 | NM_017866.5 |  |  |  | x |  |  |
| TMEM94 | NM_014738.5 |  |  | x |  |  |  |
| TMLHE | NM_018196.3 |  |  | x |  |  |  |
| TMX2 | NM_015959.3 |  |  | x |  |  |  |
| TNFRSF1A | NM_001065.3 |  |  |  | x |  |  |
| TNK2 | NM_001010938.1 |  |  | x |  |  |  |
| TNXB | NM_019105.6 |  |  |  | x |  |  |
| TP63 | NM_003722.4 |  |  |  | x |  |  |
| TPP1 | NM_000391.3 |  |  | x |  |  |  |
| TRAF3IP1 | NM_015650.3 |  |  |  | x |  |  |
| TRAF7 | NM_032271.2 |  |  | x |  |  |  |
| TRAIP | NM_005879.2 |  |  |  | x |  |  |
| TRAP1 | NM_016292.2 |  |  |  | x |  |  |
| TRAPPC9 | NM_031466.7 |  | x | x |  |  |  |
| TREX1 | NM_033629.5 |  |  |  | x |  |  |
| TRIM32 | NM_012210.3 |  | x | x | x |  |  |
| TRIO | NM_007118.3 |  |  | x |  |  |  |
| TRIP12 | NM_004238.2 |  | x | x |  |  |  |
| TRMT10A | NM_152292.4 | x |  |  |  |  |  |
| TRMT5 | NM_020810.3 |  |  |  | x |  |  |
| TRNT1 | NM_182916.2 |  |  |  | x |  |  |
| TRPC5 | NM_012471.2 |  |  | x |  |  |  |
| TRPC6 | NM_004621.5 |  |  |  | x |  |  |
| TRPM6 | NM_017662.4 |  |  |  | x |  |  |
| TRPM7 | NM_017672.5 |  |  |  | x |  |  |
| TRPS1 | NM_014112.4 |  |  |  | x |  |  |
| TRPV5 | NM_019841.6 |  |  |  | x |  |  |
| TRPV6 | NM_018646.5 |  |  |  | x |  |  |
| TRRAP | NM_003496.3 |  |  | x |  |  |  |
| TSC1 | NM_000368.4 |  |  | x | x |  |  |
| TSC2 | NM_000548.4 |  |  | x | x |  |  |
| TSPAN7 | NM_004615.3 |  |  | x |  |  |  |
| TSR2 | NM_058163.2 |  |  |  | x |  |  |
| TTC21B | NM_024753.4 |  |  |  | x |  |  |
| TTC37 | NM_014639.3 |  |  |  | x |  |  |
| TTC8 | NM_198309.3 |  | x | x | x |  |  |
| TTR | NM_000371.3 |  |  |  | x |  |  |
| TUB | NM_003320.4 |  | x |  |  |  |  |
| TUBA1A | NM_006009.3 |  |  | x |  |  |  |
| TUBB2B | NM_178012.4 |  |  | x |  |  |  |
| TUBGCP2 | NM_001256617.1 |  |  | x |  |  |  |
| TUSC3 | NM_006765.3 |  |  | x |  |  |  |
| TWIST1 | NM_000474.3 |  |  | x |  |  |  |
| TWIST2 | NM_057179.2 |  |  |  | x |  |  |
| TXNL4A | NM_006701.4 |  |  |  | x |  |  |
| UBE2A | NM_003336.3 |  | x | x |  |  |  |
| UBE2T | NM_014176.3 |  |  |  | x |  |  |
| UBE3A | NM_130838.2 |  | x | x |  |  |  |
| UBE3B | NM_130466.3 |  |  | x |  |  |  |
| UBR1 | NM_174916.2 |  |  | x | x |  |  |
| UCP2 | NM_003355.2 | x |  |  |  |  |  |
| UCP3 | NM_003356.3 |  | x |  |  |  |  |
| UMOD | NM_003361.3 |  |  |  | x |  |  |
| UMPS | NM_000373.3 |  |  |  | x |  |  |
| UPB1 | NM_016327.2 |  |  | x | x |  |  |
| UPF3B | NM_080632.2 |  |  | x |  |  |  |
| UPK3A | NM_006953.3 |  |  |  | x |  |  |
| UQCC2 | NM_032340.3 |  |  |  | x |  |  |
| UQCRFS1 | NM_006003.2 |  |  | x |  |  |  |
| UROC1 | NM_144639.2 |  |  | x |  |  |  |
| USP27X | NM_001145073.2 |  |  | x |  |  |  |
| USP9X | NM_001039590.2 |  |  | x | x |  |  |
| VANGL1 | NM_138959.2 |  |  |  | x |  |  |
| VCP | NM_007126.4 |  |  | x |  |  |  |
| VDR | NM_001017535.1 |  |  |  | x |  |  |
| VHL | NM_000551.3 |  |  |  | x |  |  |
| VIPAS39 | NM_022067.3 |  |  |  | x |  |  |
| VLDLR | NM_003383.4 |  |  | x |  |  |  |
| VPS13B | NM_017890.4 |  | x | x |  |  |  |
| VPS33B | NM_018668.4 |  |  |  | x |  |  |
| VPS4A | NM_013245.3 |  |  | x |  |  |  |
| WAC | NM_016628.4 |  |  | x |  |  |  |
| WAS | NM_000377.2 |  |  |  | x |  |  |
| WASF1 | NM_003931.2 |  |  | x |  |  |  |
| WDPCP | NM_015910.6 |  | x | x | x |  |  |
| WDR19 | NM_025132.3 |  |  |  | x |  |  |
| WDR34 | NM_052844.3 |  |  |  | x |  |  |
| WDR35 | NM_001006657.1 |  |  |  | x |  |  |
| WDR37 | NM_014023.3 |  |  | x |  |  |  |
| WDR45 | NM_007075.3 |  |  | x |  |  |  |
| WDR45B | NM_019613.3 |  |  | x |  |  |  |
| WDR60 | NM_018051.4 |  |  |  | x |  |  |
| WDR62 | NM_001083961.1 |  |  | x |  |  |  |
| WDR72 | NM_182758.3 |  |  |  | x |  |  |
| WDR73 | NM_032856.3 |  |  |  | x |  |  |
| WFS1 | NM_006005.3 | x |  |  | x |  |  |
| WNK1 | NM_018979.3 |  |  |  | x |  |  |
| WNK3 | NM_020922.4 |  |  | x |  |  |  |
| WNK4 | NM_032387.4 |  |  |  | x |  |  |
| WNT3 | NM_030753.4 |  |  |  | x |  |  |
| WNT4 | NM_030761.4 |  |  |  | x |  |  |
| WNT5A | NM_003392.4 |  |  |  | x |  |  |
| WNT7A | NM_004625.3 |  |  |  | x |  |  |
| WT1 | NM_024426.5 |  |  |  | x |  |  |
| WWOX | NM_016373.3 |  |  | x |  |  |  |
| XDH | NM_000379.3 |  |  |  | x |  |  |
| XPNPEP3 | NM_022098.3 |  |  |  | x |  |  |
| XPO5 | NM_020750.2 |  |  |  | x |  |  |
| XRCC4 | NM_022406.3 |  |  |  | x |  |  |
| XYLT2 | NM_022167.3 |  |  |  | x |  |  |
| YAP1 | NM_001130145.2 |  |  | x | x |  |  |
| YIPF5 | NM_001024947.3 | x |  | x |  |  |  |
| YWHAE | NM_006761.4 |  |  | x |  |  |  |
| YY1AP1 | NM_001198903.1 |  |  |  | x |  |  |
| ZAP70 | NM_001079.3 |  |  |  | x |  |  |
| ZBTB18 | NM_205768.2 |  |  | x |  |  |  |
| ZBTB20 | NM_001164342.2 |  |  | x |  |  |  |
| ZC3H14 | NM_024824.4 |  |  | x |  |  |  |
| ZC4H2 | NM_018684.3 |  |  | x |  |  |  |
| ZDHHC15 | NM_144969.2 |  |  | x |  |  |  |
| ZDHHC9 | NM_016032.3 |  |  | x |  |  |  |
| ZEB2 | NM_014795.3 |  |  | x |  |  |  |
| ZFHX4 | NM_024721.4 |  |  | x |  |  |  |
| ZFP57 | NM_001109809.2 | x |  |  |  |  |  |
| ZIC3 | NM_003413.3 |  |  |  | x |  |  |
| ZMIZ1 | NM_020338.3 |  |  | x |  |  |  |
| ZMPSTE24 | NM_005857.4 |  |  |  | x |  |  |
| ZMYM2 | NM_003453.4 |  |  |  | x |  |  |
| ZMYM3 | NM_201599.2 |  |  | x |  |  |  |
| ZMYND11 | NM_006624.5 |  |  | x |  |  |  |
| ZNF292 | NM_015021.2 |  |  | x |  |  |  |
| ZNF423 | NM_015069.4 |  |  |  | x |  |  |
| ZNF526 | NM_133444.2 |  |  | x |  |  |  |
| ZNF687 | NM_020832.2 |  |  |  | x |  |  |
| ZNF711 | NM_021998.4 |  |  | x |  |  |  |
| ZNF81 | NM_007137.3 |  |  | x |  |  |  |
